# Classifying gastric cancer using FLORA reveals clinically relevant molecular subtypes and highlights *LINC01614* as a biomarker for patient prognosis

**DOI:** 10.1038/s41388-021-01743-3

**Published:** 2021-03-19

**Authors:** Yiyun Chen, Wing Yin Cheng, Hongyu Shi, Shengshuo Huang, Huarong Chen, Dabin Liu, Weiqi Xu, Jun Yu, Jiguang Wang

**Affiliations:** 1 Division of Life Science and Department of Chemical and Biological Engineering, Center of Systems Biology and Human Health, State Key Laboratory of Molecular Neuroscience, The Hong Kong University of Science and Technology, Hong Kong SAR, China; 2 Institute of Digestive Disease and The Department of Medicine and Therapeutics, State Key Laboratory of Digestive Disease, Li Ka Shing Institute of Health Sciences, CUHK Shenzhen Research Institute, The Chinese University of Hong Kong, Hong Kong SAR, China; 3 Department of Hepatic Surgery, Fudan University Shanghai Cancer Center, Department of Oncology, Shanghai Medical College, Fudan University, Shanghai, China; 4 Hong Kong Center for Neurodegenerative Diseases, Hong Kong Science Park, Hong Kong SAR, China

**Keywords:** Gene expression profiling, Transcriptomics, Prognostic markers

## Abstract

Molecular-based classifications of gastric cancer (GC) were recently proposed, but few of them robustly predict clinical outcomes. While mutation and expression signature of protein-coding genes were used in previous molecular subtyping methods, the noncoding genome in GC remains largely unexplored. Here, we developed the fast long-noncoding RNA analysis (FLORA) method to study RNA sequencing data of GC cases, and prioritized tumor-specific long-noncoding RNAs (lncRNAs) by integrating clinical and multi-omic data. We uncovered 1235 tumor-specific lncRNAs, based on which three subtypes were identified. The lncRNA-based subtype 3 (L3) represented a subgroup of intestinal GC with worse survival, characterized by prevalent *TP53* mutations, chromatin instability, hypomethylation, and over-expression of oncogenic lncRNAs. In contrast, the lncRNA-based subtype 1 (L1) has the best survival outcome, while *LINC01614* expression further segregated a subgroup of L1 cases with worse survival and increased chance of developing distal metastasis. We demonstrated that *LINC01614* over-expression is an independent prognostic factor in L1 and network-based functional prediction implicated its relevance to cell migration. Over-expression and CRISPR-Cas9-guided knockout experiments further validated the functions of *LINC01614* in promoting GC cell growth and migration. Altogether, we proposed a lncRNA-based molecular subtype of GC that robustly predicts patient survival and validated *LINC01614* as an oncogenic lncRNA that promotes GC proliferation and migration.

## Introduction

The classification of gastric cancer (GC) is essential for addressing the inter-tumoral heterogeneity, predicting the clinical outcome, and customizing treatments for different patients. Methods based on histological or molecular characteristics of GC have been proposed. Lauren classification, which is based on tumor histology, divides GC into diffuse or intestinal subtypes. Diffuse-type GC is generally more aggressive [1], yet the association between Lauren classification and prognosis is debated in different studies. Molecular subtype systems were proposed by The Cancer Genome Atlas (TCGA) [2, 3], dividing GC into Epstein-Barr-virus-positive (EBV), microsatellite-instable (MSI), genomically-stable (GS), chromosomal-instable (CIN), and hypermutated-single-nucleotide-variants (HM-SNV) subtypes. However, similar survival outcomes were observed in different subtypes. Alternative molecular classification systems (systematically reviewed by Serra et al. [4]) have identified expression (GS expression signatures [5] and mesenchymal phenotype [6, 7]), EBV status, and mutational signatures [8, 9] that predict worse survival. Overall, current molecular classification methods focus merely on mutations or dysregulated expression of coding genes, while the noncoding genome of GC is largely unexplored.

In recent years, lncRNAs (defined as non-coding RNAs longer than 200 nucleotides) have been extensively studied, revealing their diverse functions in tumor progression, metastasis, and drug resistance [10]. Compared to coding genes, the lncRNA expression level is generally lower and tends to be more spatiotemporally specific [11, 12]. Given the ability of lncRNAs to reflect minor changes in cell states, we hypothesize the potential capability of lncRNAs in tumor subtype classification. As the current lncRNA annotation catalog is not complete, through ab initio assembly, it is possible to find previously unknown lncRNAs with important functions. LncRNA analysis pipelines including ATRAIN [13], UClncR [14], and NORI [15] have incorporated the transcript assembly step into their workflows. However, no efficient functional prediction methods have been adopted. The assembly process also becomes increasingly time-consuming with the increasing sequencing depth and expanding sample collections.

In this study, we developed fast long-noncoding RNA analysis (FLORA), an efficient computational pipeline for lncRNA identification, characterization, and functional prediction. From the whole transcriptome sequencing data of 375 GC tumor samples and 32 tumor-adjacent samples in TCGA, we identified 1547 novel lncRNAs as well as 3153 annotated lncRNAs that are expressed across tumor samples. Subsequently, three molecular subtypes of GC were identified based on 1235 tumor-specific lncRNAs, and the lncRNA-based GC subtype 3 (L3) exhibited elevated expression of 359 lncRNAs and worse prognosis independent of tumor stage, age, and cohorts. Surprisingly, most of the L3 cases were labeled intestinal, a histologic subtype that generally associates with a better prognosis. In addition, the L3 subtype was also featured by the high prevalence of *TP53* mutations, chromatin instability, and genome-wide hypomethylation. Genetic and epigenetic alterations both potentially contribute to the elevated expression of oncogenic lncRNAs such as *H19* in the L3 subtype. While most of the prognostic lncRNAs were specifically expressed in the L3 subtype, *LINC01614* has the highest expression level in the L1 subtype, which has the best survival outcome. Interestingly, a subgroup of L1 cases over-expressing *LINC01614* demonstrated worse survival as well as a higher risk of developing metastatic tumor. FLORA predicted the function of *LINC01614* in mediating extracellular matrix (ECM) remodeling and cell migration, which was further validated by overexpression and CRISPR-Cas9 knockout experiments in GC cell lines. In summary, our study identified lncRNA-based molecular subtypes that robustly predict patient survival and revealed the functions of *LINC01614* in promoting cell proliferation and migration, which have great potentials in the future development of GC biomarkers.

## Results

### Identification of oncogenic lncRNAs in GC using the FLORA workflow

Examining reads mapped to different genomic regions, we identified on average 2.9% of reads are properly mapped and derived from intergenic regions that could potentially encode lncRNAs (Fig. S1A, B). Motivated by the observation, we developed FLORA to efficiently reconstruct the noncoding transcriptome and prioritize cancer-relevant lncRNAs (Fig. S1C). FLORA started by pre-processing the alignment results and eliminating reads mapped to coding regions and other RNA species, which greatly reduce the data size and accelerate transcript assembly. After transcriptome assembly and merging, a transcript will be reported as a prospective lncRNA if it passes coding potential assessment, has a desirable length (over 200 bases), and at least two exons. The function of each lncRNA is then predicted via the construction of co-expression networks, followed by gene ontology (GO) enrichment analysis.

FLORA identified 28,507 loci that potentially encode lncRNAs from TCGA GC and normal samples (Table S1, Supplementary Materials and Methods). Among all the lncRNA-encoding genes reported by FLORA, 13,675 loci overlapped with annotated lncRNAs, such as *H19* and *PVT1*, while 10,356 loci were not present in GENCODE [12], Ensembl [16] or RefSeq [17] annotation and were thus considered novel (Fig. S1D). Finally, we kept 4700 lncRNAs with mean expression level over 0.1 FPKM for downstream analysis, including 1547 novel and 3153 known lncRNAs in GENCODE (Table S2). Interestingly, the fraction of reads from known and potential lncRNAs was significantly higher in tumors compared to their paired normal samples, as well as across all samples (Fig. S1E, F). In contrast, the expression from coding regions in tumor and normal tissue was less divergent (Fig. S1G), suggesting lncRNAs exhibit tumor-specific expression patterns. Compared to normal gastric tissues, 1235 lncRNAs were significantly upregulated and 689 were downregulated in GC samples (Table S3), including several well-characterized oncogenic lncRNAs such as *H19* [18], *HOTAIR* [19], and *DUXAP8* [20].

### Clinical relevance of lncRNA-based GC subtypes

**Fig. 1:**
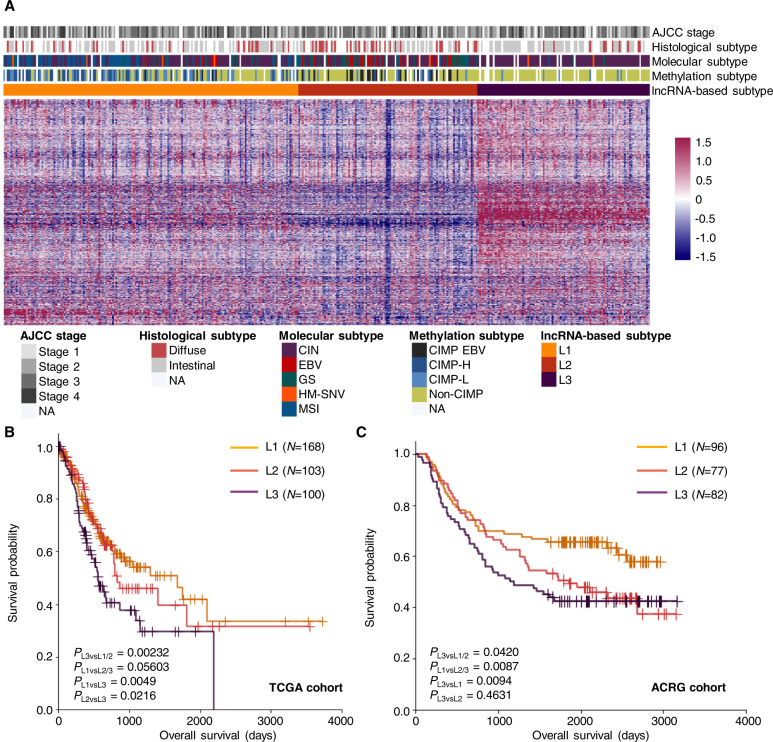
Molecular classification based on tumor-specific lncRNA expression defined novel GC subtypes with prognostic value. **A** Heatmap of differentially expressed lncRNAs in three GC subtypes. Each column represented one GC sample, and each row represented a lncRNA. The AJCC stage, histology, molecular subtype, and methylation-based subtype for each sample were marked on the top panel, with a white grid representing non-available (NA) information. **B**, **C** Kaplan–Meier curve illustrating the overall survival for GC patients of the three subtypes from the TCGA (**B**) and ACRG (**C**) cohort. *P*-values were calculated by log-rank test.

As lncRNA expression is highly dynamic and sensitive to changes in cell states, we examined the applicability of lncRNA-based classification in separating GC cases. Based on 1235 GC-specific lncRNAs, we identified three robust clusters from 375 TCGA GC samples: L1 (*N* = 171), L2 (*N* = 104), and L3 (*N* = 100) (Fig. 1A, Fig. S2A–C and Table S1). 359 lncRNAs were specifically enriched in the L3 subtype (Table S3), including the oncogenic lncRNAs *DUXAP8* [20], *H19* [21], and *HOXC-AS3* [22], while the tumor suppressor *GUARDIN* [23] was downregulated (Fig. S2D–G). Intriguingly, the L3 subtype was associated with the worst overall survival, while the L1 subtype has the best survival outcome (Fig. 1B). To validate the clinical relevance of lncRNA-based subtypes, we analyzed the microarray data from GC cases collected by the Asian Cancer Research Group (ACRG) [6], Yonsei University [24], and Singapore National Cancer Center [25, 26], and classified the independent cohorts into three lncRNA-based subtypes by support vector machine model (Table S4–6). Consistently, the L3 subtype showed poor overall survival in all three independent cohorts (Figs. 1C and S3A, B), demonstrating the robustness of lncRNA-based GC subtyping across populations and profiling platforms.

**Fig. 2:**
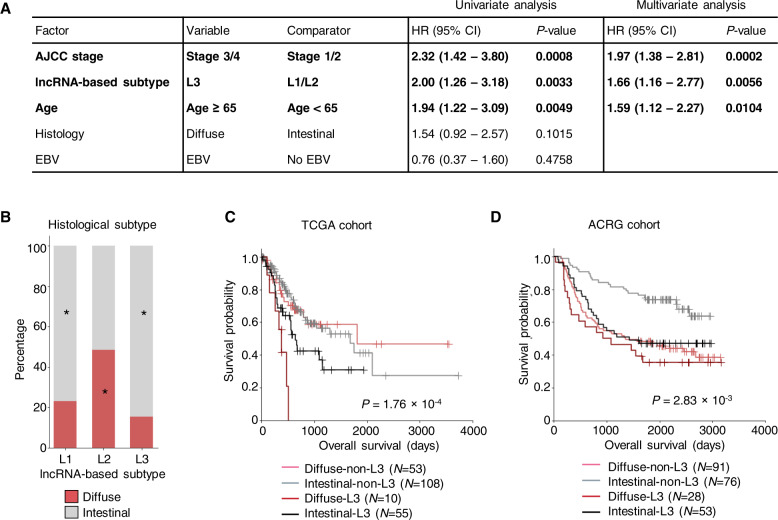
LncRNA-based subtype L3 represents a subset of intestinal GC with a poor prognosis. **A** Value and 95%-confidence interval (95% CI) of the hazard ratio (HR) of different factors considered in the univariate and multivariate Cox regression analysis of the TCGA cohort. AJCC the American Joint Committee on Cancer. **B** Distribution of intestinal and diffuse tumors in lncRNA subtypes in the 375 TCGA GC samples. **C**, **D** Survival probability of TCGA (**C**) and ACRG (**D**) patients segregated by the combination of histology and lncRNA-based subtypes. The *P*-values were calculated by multivariate log-rank test.

While the stage of GC (determined by the American Joint Committee on Cancer (AJCC)) and the age at diagnosis were also significantly associated with survival, the lncRNA-based subtypes were independent of the two variables (Fig. S3C, D). Multivariable Cox regression analysis revealed that the L3 subtype, as well as tumor stage and age at initial diagnosis, was a predictor of worse prognosis in GC (Fig. 2A). The combination of tumor stage and lncRNA-based subtype yielded greater predictive power (*P* = 4.78 × 10^−5^ by multivariate log-rank test) compared to considering individual factors alone (*P* = 2.3 × 10^−4^ and 1.4 × 10^−2^, for stage and lncRNA-based subtype, respectively) (Fig. S3E). Notably, among the stage I/II tumor cases, the L3 subtype demonstrated significantly worse survival than L1 and L2 subtype (*P* = 0.016 by log-rank test), suggesting lncRNA-based subtype can identify high-risk GC cases for early intervention. In concordance with the analysis of the TCGA dataset, the L3 subtype was identified as an independent prognosis factor in independent cohorts (Figs. S3F–H, S4A, B), demonstrating the robustness of lncRNA-based subtype in predicting survival outcome.

### Associations between lncRNA-based subtypes and histology

The histological subtypes (or Lauren classification) of GC has long been considered as a prognostic marker, with the diffuse subtype associated with poor survival and the intestinal subtype with better outcome [1]. Although consistent conclusions can be drawn from the cases in the ACRG cohort, no significant difference in patient survival was observed in patients from TCGA or Singapore cohort (Fig. S4C–E). Therefore, the prediction of clinical outcomes solely based on the Lauren classification may yield poor accuracy. Comparing Lauren classification and lncRNA-based subtypes, we observed enrichment of the diffuse-type GC in L2, while the intestinal-type is enriched in L1 and L3 (Figs. 2B and S4F, G). However, the intestinal-type GC demonstrated a vast difference in survival, with the intestinal-L3 subgroup demonstrating a worse prognosis than the intestinal-non-L3 subgroup (Fig. 2C, D). Collectively, our analysis demonstrated that the lncRNA-based L3 subtype further distinguished a subset of intestinal histology with adverse survival outcomes across cohorts, which were overlooked in previous studies.

### Genomic and epigenomic characteristics of lncRNA-based GC subtypes

**Fig. 3:**
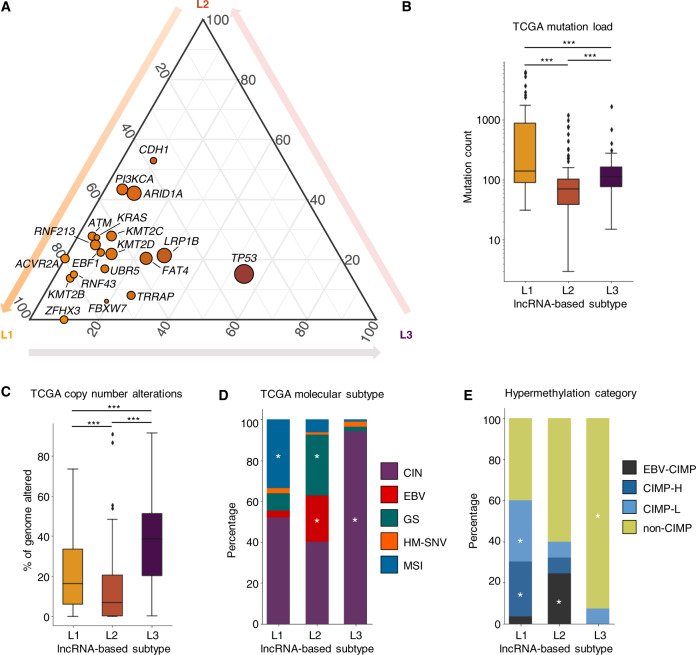
Characterization of mutations, copy number variations, and epigenetic alterations in lncRNA-based GC subtypes. **A** Frequency of somatic mutations that were significantly enriched in at least one lncRNA-based subtype (Fisher’s exact test, *P* < 0.001). Each dot represented one frequently mutated gene in GC, with size proportional to its overall mutation frequency. **B** Somatic mutation load in different lncRNA-based subtypes. ***P < 0.001 by unpaired two-tailed *t*-test. **C** The fraction of genome with copy number alterations in different lncRNA-based subtypes. ***P < 0.001 by unpaired two-tailed *t*-test. **D** Distribution of TCGA molecular subtypes in the three lncRNA-based subtypes. **E** Percentage of L1, L2, and L3 subtype samples in the EBV-CIMP, CIMP-H, CIMP-L, and non-CIMP categories. *Represented enrichment with *P* < 0.05 by Fisher’s exact test.

To characterize the genomic features for each lncRNA-based subtype, we integrated mutation and CNV profiles of GC in the TCGA and ACRG cohorts. Among the most common mutations in GC, *TP53* mutations were significantly enriched in the L3 subtype from both the TCGA and ACRG cohort (Figs. 3A and S5A), while *ARID1A*, *PIK3CA*, *KMT2B*, *KRAS*, and *FBXW7* mutations were more frequent in the L1 subtype. Mutations in *CDH1* were more common in the L2 subtype, which was consistent with previous studies that the diffuse-type GC frequently harbors dysfunctional *CDH1* [27]. In addition, higher mutation load was observed in the L1 subtype, while chromosomes of L3 subtype samples were more unstable and carried significantly more CNV than other subtypes (Figs. 3B, C and S5B–E). Compared to L1 and L2 subtypes, 19q12(*CCNE1*), 17q12(*ERBB2*), 7p11.2(*EGFR*), and 20q13.2(*ZNF217*) were more frequently amplified in the L3 subtype, while 6p25.3(*FOXC1*) was frequently deleted (Fig. S5F–I). Among the frequently amplified oncogenes in L3, *CCNE1* exhibited a significantly higher expression level in L3 (Fig. S5J).

While these molecular features were utilized for GC classification, limited predictive power has been provided by molecular subtypes proposed by the TCGA study [2, 3] (Fig. S5K, L). We found that L3 was mostly comprised of CIN tumor (96%), L2 contained a higher proportion of EBV and GS tumor, while L1 was enriched with MSI tumor (Figs. 3D and S5M). Although both contained a large fraction of CIN-subtype GC, L1-CIN and L3-CIN subgroups demonstrated a vast difference in patient survival (Fig. S5N). Therefore, substantial heterogeneities were observed in previous subtyping methods, hindering the prediction of clinical outcomes.

Apart from the genomic alterations, the L3 subtype also exhibitied a distinct DNA methylation profile. Based on the level of CpG island methylator phenotype (CIMP), gastrointestinal tumors were classified into EBV-CIMP (EBV-associated-CIMP), CIMP-H (high-CIMP), CIMP-L (low-CIMP), and non-CIMP [3]. The non-CIMP category was highly enriched in the L3 subtype (93%) (Fig. 3E). The expression levels of *TET1*, *TET3*, and *TDG*, all of which are involved in the DNA demethylation pathways, were significantly upregulated in the L3 subtype compared with the non-L3 samples (Fig. S6A, B), which may partially explain the non-CIMP methylation phenotype in L3 subtype. Moreover, epigenomic regulations may play important roles in generating the unique lncRNA expression profile of the L3 subtype.

### Interplay of *TP53* mutations and DNA demethylation drives oncogenic lncRNA expression and aggressive phenotype in L3 subtype

Out of the L3-enriched lncRNAs, 137 demonstrated hypomethylation in the L3 subtype, including *H19*, *HOXC13-AS*, *NOVA1-AS1*, and *DUXAP8* (Fig. S6C–F and Table S3). The methylation level of *H19* was negatively correlated with *H19* expression, as well as the level of *TET1*, *TET3*, and *TDG* (Fig. S6G), suggesting epigenetic regulations contribute to the lncRNA over-expression phenomenon in the L3 subtype. Moreover, as a guardian of the genome, p53 is involved in the regulation of the expression of various lncRNAs [28], including suppression of *H19* promoter activity [29]. In the TCGA dataset, *H19* expression and methylation were both significantly altered in GC samples with *TP53* mutation (Fig. S6H, I). Therefore, we propose that mutations in *TP53* and genome-wide hypomethylation in the L3 subtype may contribute to the hyper-expression of several L3-enriched lncRNA species (Table S3).

Out of the lncRNAs with elevated expression and hypomethylation in the L3 subtype, *H19* is a molecular marker for GC diagnosis [18, 30] and plays important functions in promoting GC cell proliferation, metastasis [31, 32], DNA hypomethylation [33], genome instability and suppression of p53 activation [21, 34]. Applying the functional prediction module of FLORA, we identified that the co-expression network of *H19* was associated with ECM organization and cell migration, which is in line with previous reports. The network-based lncRNA function prediction module was also validated on other well-characterized lncRNAs such as *HOTAIR* [35] and *HOTTIP* [36], where FLORA successfully reconstructed the co-expression of *HOTAIR* with *HOXA* and *HOXD* gene clusters, and *HOTTIP* with *HOXA13*, *HOXA11*, and *HOXA10* (Fig. S7). Therefore, through the combination of *TP53* mutation and genome-wide hypomethylation, oncogenic lncRNAs including *H19* were highly expressed in the L3 subtype, leading to more aggressive disease and worse patient survival.

In summary, using GC-specific lncRNAs as features, we identified three lncRNA-based GC subtypes with significant associations with survival outcome and characterized their histological and molecular characteristics (Fig. S8A). The most aggressive L3 subtype demonstrated enrichment of intestinal histology, *TP53* mutations, chromatin instability, genome-wide hypomethylation, and over-expression of a large number of lncRNA, including several well-known oncogenic lncRNA species. In contrast, L1 was enriched with intestinal histology and MSI, and L2 was enriched with diffuse histology, EBV-positive, and GS tumor. Classifying GC patients in independent cohorts by SVM models also revealed the robustness and applicability of lncRNA-based subtypes in predicting patient survival.

### Identification of oncogenic lncRNAs in GC

**Fig. 4:**
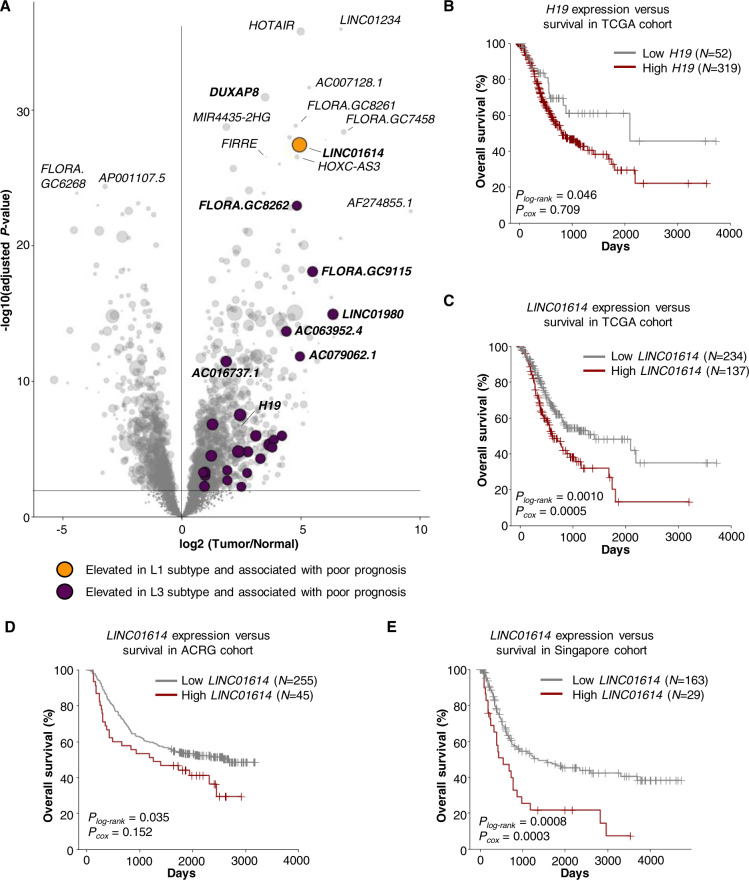
Identification and prioritization of noncoding prognostic markers for gastric cancer. **A** Volcano plot of differentially expressed lncRNAs between tumor and normal samples in TCGA. The lncRNAs that were significantly upregulated in L3 and L1 were highlighted and colored purple and yellow, respectively. The lncRNAs that were upregulated in tumors and correlated with unfavorable prognosis were enlarged, with dot size proportional to −log10(*P*-value) of Cox regression test. **B** Expression of *H19* was associated with unfavorable prognosis in the TCGA cohort. **C**–**E** Expression of *LINC01614* was associated with unfavorable prognosis in the TCGA (**C**), ACRG (**D**), and Singapore cohort (**E**). The cut-off for defining the high expression group and low expression group were selected to maximize the survival difference (with the minimum *P*-value by log-rank test (*P*_*log-rank*_) of all cut-off values tested). *P*_*cox*_ was calculated by the Cox regression test.

Out of the 1235 lncRNAs with significantly elevated expression in GC compared to tumor-adjacent normal samples (Fig. 4A), we identified several lncRNAs that were associated with worse survival outcomes, including the GC biomarker *H19* [18] and other less-studied species such as *LINC01614* (Fig. 4B and Table S3). Using an ensemble approach that considers various parameters, including lncRNA expression in tumors, association with patient survival, adjacency to GC-risk-associated SNPs, and evidence from the literature, we prioritized 50 potential candidates that may function as GC biomarkers. *LINC01614* was identified as the most promising lncRNA biomarker (Fig. S8B and Table S7) and its association with GC was validated in ACRG and Singapore cohort (Fig. 4D, E). Interestingly, most of the prognosis-related lncRNAs were over-expressed in the L3 subtype, which was characterized by poor survival outcome, high frequency of *TP53* mutations, chromatin instability, and genome-wide hypomethylation. However, *LINC01614* showed significantly higher expression in the L1 subtype and the MSI subtype (Figs. 4A and S8C–F).

**Fig. 5:**
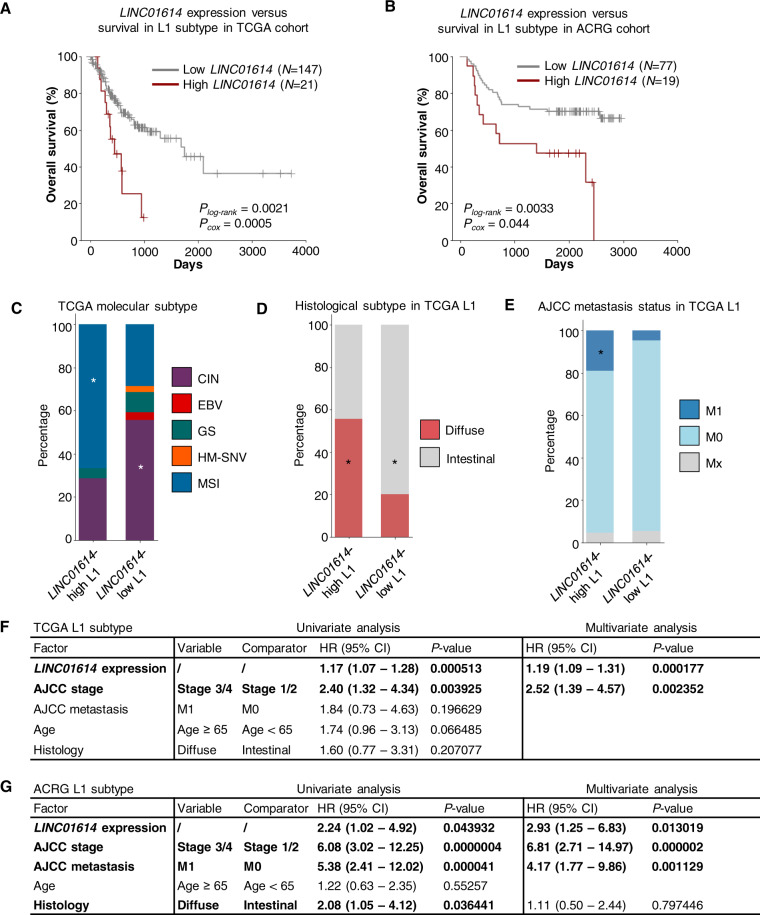
*LINC01614* stratified lncRNA-based subtype L1 into patient clusters with different survival outcomes. **A**, **B** High expression of *LINC01614* in L1 subtype was associated with unfavorable prognosis in TCGA (**A**) and ACRG (**B**) cohorts. The cut-off for defining the high expression group and low expression group were selected to maximize the survival difference (with the minimum *P*-value by log-rank test (*P*_*log-rank*_) of all cut-off values tested). *P*_*cox*_ was calculated by the Cox regression test. **C**–**E** Distribution of TCGA molecular subtypes (**C**), histological subtype (**D**), and metastasis status (**E**) in L1 patients with high and low expression of *LINC01614*. *Enrichment with *P* < 0.05 by Fisher’s exact test. **F**, **G** Hazard ratio (HR) of different factors considered in the univariate and multivariate cox regression analysis of TCGA cohort (**F**) and ACRG cohort (**G**). The significant factors were highlighted in bold.

As the L1 subtype was found to associate with the best survival outcome (Fig. 1B, C), we analyzed whether *LINC01614* can further segregate the L1 subtype and better predict patient survival. Indeed, elevated expression of *LINC01614* was also associated with poor survival in the L1 subtype (Fig. 5A, B). The subgroup of L1 cases with high expression of *LINC01614* has worse survival outcome, enrichment of MSI, higher frequency of the diffuse histology, and an increased chance of developing distal metastasis compared to the *LINC01614*-low L1 cases in the TCGA cohort (Fig. 5C–E). While MSI and diffuse histology were also enriched in the *LINC01614*-high L1 subgroup in the ACRG cohort, the chance of metastasis was not significantly different in *LINC01614*-high versus *LINC01614*-low L1 subgroup (Fig. S9A–C). Nevertheless, *LINC01614* expression was an independent prognostic factor in the L1 subtype of both the TCGA and ACRG cohort (Fig. 5F, G). L1 subtype was consisted of around 40% of GC cases and was relatively heterogeneous, and *LINC01614* can robustly segregate a subgroup of L1 patients with worse survival.

Therefore, *LINC01614* robustly predicted survival outcome and potentially contribute to GC metastasis. We applied the functional prediction module and revealed over 100 genes that were significantly associated with *LINC01614* (Fig. S9D). Moreover, a large fraction of genes in the co-expression network of *LINC01614* were involved in ECM organization (Fig. S9E), suggesting *LINC01614* is functionally involved in regulating ECM remodeling and promoting cell migration.

### Experimental validation of *LINC01614* in promoting GC

**Fig. 6:**
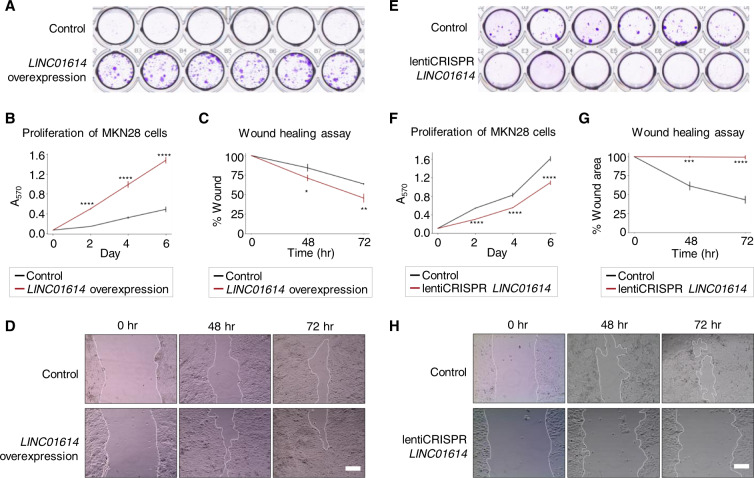
Validation of oncogenic roles of *LINC01614* in gastric cancer. **A** Colony formation capability of MKN28 cell line with and without *LINC01614* overexpression. **B**, **C** Proliferation rate (**B**) and migration capability (**C**) under *LIN01614* overexpression (red) and control (black) in MKN28 cell line. A570: absorbance at 570 nm. **D** Representative images of control or *LINC01614*-overexpressed MKN28 cells migrating into the wounded areas at 0 h, 48 h, and 72 h. The scale bar indicates 20 µm. **E** Colony formation capability of MKN28 cell line transfected with CRISPR-Cas9 vector for *LINC01614* knockout and with control vector. **F**, **G** Proliferation rate (**F**) and migration capability (**G**) in MKN28 cells with *LINC01614* knockout (red) and control (black). **H** Representative images of control or *LINC01614*-knockout MKN28 cells migrating into the wounded areas at 0 h, 48 h, and 72 h. The scale bar indicates 20 µm. In all experiments, three biological replicates were performed for each group. Data are represented as mean ± SD. *P < 0.05; **P < 0.01; ***P < 0.001; ****P < 0.0001 by two-tailed unpaired *t*-test.

Using semi-quantitative PCR, we validated that *LINC01614* is highly expressed in GC cell lines and low in normal cells (GSE1) (Fig. S10A). Ectopic expression of *LINC01614* in MKN28, MKN1, GES1, and MGC803 cell lines resulted in accelerated cell proliferation, colony formation, and migration (Figs. 6A–D and S10B–M). Furthermore, we performed CRISPR-Cas9 knockout of *LINC01614* in MKN28, MKN1, and GES1 cells and observed attenuated cell proliferation, defects in colony formation, and significantly reduced migration in wound healing assay (Figs. 6E–H and S11), supporting the oncogenic role of *LINC01614* in promoting GC cell growth and migration. Recent studies have shown that *LINC01614* is implicated in lung cancer [37, 38] and oesophageal squamous cell carcinoma [39], and our study further revealed the oncogenic functions of *LINC01614* in GC and suggested potentials of *LINC01614* as a biomarker of GC.

To examine the downstream effectors of *LINC01614*, we generated RNA-seq data of the GC cell lines after *LINC01614* CRISPR-Cas9 knockout or over-expression. Over-expression of *LINC01614* in GES1 and MGC803 cell lines resulted in the upregulation of 234 genes, including *SERPINE1*, *LAMC2*, and *STC2*. A significant portion (63.2%) of the up-regulated genes was also identified by the co-expression network of *LINC01614* generated using the TCGA dataset (Fig. S12A, B). In contrast, after *LINC01614* knockout, 227 genes were downregulated in both the MKN1 and GES1 cell lines, among which 158 genes (69.6%) overlapped with genes in the co-expression network of *LINC01614* (Fig. S12C, D). Pre-ranked gene-set enrichment analysis also revealed pathways that were strongly correlated with *LINC01614* manipulation, including epithelial-mesenchymal transition, MYC targets, unfolded protein response, IL2/STAT5 signaling, KRAS signaling, and TNFA signaling pathways (Fig. S12E and Table S8), which is consistent with the functions of *LINC01614* in promoting cell proliferation and migration. Combining the co-expression network of *LINC01614* and transcriptome data collected before and after *LINC01614* manipulation, 520 and 28 genes were identified to have a positive and negative association with *LINC01614*, respectively (Table S9). Altogether, our study revealed the oncogenic functions of *LINC01614* in GC and identified the downstream genes that are potentially regulated by *LINC01614* through direct or indirect mechanisms.

## Discussion

The recent explosive growth of whole transcriptome sequencing data has provided valuable resources for the discovery of a comprehensive set of disease-associated lncRNAs. To build an ideal tool for the big data, we constructed the FLORA pipeline, which by removing reads mapped to the coding regions early in the workflow, can accelerate the process of transcript assembly and lncRNA identification. Considering that most of the whole transcriptomic data are not strand-specific, the accurate reconstruction of lncRNA species that overlap with known coding genes is challenging. Thus, although the prefiltering step excludes reads derived from potential antisense and intronic lncRNA species, it also reduces the chance of false discovery and aids the identification of novel intergenic lncRNAs. In addition, as lncRNA with high expression often yields better reconstruction, we further filtered the list of novel lncRNAs by expression level to select candidates with high confidence. Applying the pipeline to 407 samples collected by TCGA, we discovered 1924 lncRNAs with dysregulated expression in GC, among which 547 have not been previously annotated. In addition to the previously reported oncogenic lncRNAs, several novel lncRNAs also exhibited tumor-specific expression patterns and were associated with poor survival, such as *FLORA.GC7366* and *FLORA.GC1247*, suggesting they may exert oncogenic functions in GC. We believe that FLORA would be a useful tool in lncRNA analysis, especially with the increasing amount of data collected by large international consortiums such as TCGA.

The capability of lncRNAs to reflect subtle changes in cellular states inspired us to identify subtypes of GC by lncRNA expression. Based on the differentially expressed lncRNAs, three GC subtypes were identified, and the low expression levels of lncRNAs did not hinder the robustness of the subtypes. The power of the L3 subtype in identifying high-risk GC cases was robust across tumor stages, cohorts, and expression profiling platforms. While other histological and molecular features, including the Lauren classification [1] and EBV status [8, 9], have been implicated as prognostic factors in GC, they do not robustly correlate with survival across cohorts. Aided by lncRNA-based subtyping, our analysis further revealed L3 as a subgroup of intestinal-type GC with a similar survival outcome as the diffuse-type GC, while most of the aggressive GC subtypes identified by previous studies are enriched with diffuse histology. Thus, our analysis revealed heterogeneity in previous molecular and histological subtypes and highlighted lncRNA-based subtypes as a robust, novel, and independent prognostic marker for GC.

Our study further illustrated the molecular alterations in the L3 subtype, which was featured by the over-expression of 359 lncRNAs, enrichment of *TP53* mutations, chromatin instability, and low frequency of CIMP. Several L3-specific lncRNAs have been characterized as oncogenic lncRNAs, including the oncofetal lncRNA *H19*. The over-expression of oncogenic lncRNAs including *H19* is associated with *TP53* mutations and hypomethylation, and *H19* promotes cell proliferation, metastasis [31, 32], DNA hypomethylation [33], genome instability, and suppression of p53 activation [21, 34], and thus contribute to the aggressive disease.

*LINC01614* was also highlighted in our analysis as a biomarker for GC diagnosis and prognosis, and its functions in promoting cell proliferation and migration were further validated in multiple GC cell lines. Future validation studies of the prognostic values of *LINC01614* in the clinical settings would be expected. Moreover, the mechanisms of *LINC01614* in GC have not been resolved to date. Recent studies illustrated the roles of *LINC01614* in promoting lung cancer cell proliferation by sequestrating miR-217 and increasing the expression of *FOXP1* [37], but the *LINC01614*/miR-217 axis has not shown significant enrichment in our analysis of the GC dataset, suggesting alternative mechanisms are involved. Combining the network biology approach and the transcriptome-wide changes in GC cell lines in response to *LINC01614* manipulation, we have curated a list of potential downstream effectors of *LINC01614* in GC and provided new insights into the functions of *LINC01614* in oncogenesis. Nevertheless, experimental validations of the regulatory relationship between *LINC01614* and its downstream targets, as well as the elucidation of the mechanism, require further investigation.

In conclusion, we developed a new computational tool FLORA for lncRNA analysis and proposed a lncRNA-based GC subtyping system that robustly segregates high-risk cases. In addition to the lncRNAs annotated in public databases, our study also curated a collection of novel lncRNAs that are highly expressed in GC. Our study comprehensively profiled the expression and clinical relevance of these GC-specific lncRNAs and identified several prognostic lncRNAs, including *LINC01614*, as potential biomarkers. The oncogenic functions of *LINC01614* were experimentally validated and its potentials as a prognostic biomarker in GC was validated in several independent cohorts. While the experimental characterization of other prognostic lncRNAs, especially the newly identified lncRNAs, has not been conducted in this study due to limited time and resource, our study has provided useful tools and resources for further investigation into the functions, mechanisms, and potential applications such as biomarkers and therapeutic targeting of lncRNAs in GC.

## Materials and methods

To comprehensively characterize the noncoding transcriptome of GC, we reanalyzed the whole transcriptome sequencing data of 407 TCGA samples, including 375 GC and 32 tumor-adjacent samples from 380 patients (Table S1) using the FLORA pipeline. The detailed description of FLORA and experiments were included in the Supplementary Materials and Methods.

## Supplementary information

Supplementary Methods

Supplementary Figures

Table S1

Table S2

Table S3

Table S4

Table S5

Table S6

Table S7

Table S8

Table S9

## References

[1] Petrelli F, Berenato R, Turati L, Mennitto A, Steccanella F, Caporale M (2017). Prognostic value of diffuse versus intestinal histotype in patients with gastric cancer: a systematic review and meta-analysis. J Gastrointest Oncol.

[2] Bass AJ, Thorsson V, Shmulevich I, Reynolds SM, Miller M, Bernard B (2014). Comprehensive molecular characterization of gastric adenocarcinoma. Nature.

[3] Liu Y, Sethi NS, Hinoue T, Schneider BG, Cherniack AD, Sanchez-Vega F (2018). Comparative molecular analysis of gastrointestinal adenocarcinomas. Cancer Cell.

[4] Serra O, Galán M, Ginesta MM, Calvo M, Sala N, Salazar R (2019). Comparison and applicability of molecular classifications for gastric cancer. Cancer Treat Rev.

[5] Sohn BH, Hwang J-E, Jang H-J, Lee H-S, Oh SC, Shim J-J (2017). Clinical significance of four molecular subtypes of gastric cancer identified by the cancer genome atlas project. Clin Cancer Res.

[6] Cristescu R, Lee JJH, Nebozhyn M, Kim K-M, Ting JC, Wong SS (2015). Molecular analysis of gastric cancer identifies subtypes associated with distinct clinical outcomes. Nat Med.

[7] Oh SC, Sohn BH, Cheong J-H, Kim S-B, Lee JE, Park KC (2018). Clinical and genomic landscape of gastric cancer with a mesenchymal phenotype. Nat Commun.

[8] Li X, Wu WKK, Xing R, Wong SH, Liu Y, Fang X (2016). Distinct subtypes of gastric cancer defined by molecular characterization include novel mutational signatures with prognostic capability. Cancer Res.

[9] He C, Qiu M, Yang X, Zhou D, Ma J, Long Y (2020). Classification of gastric cancer by EBV status combined with molecular profiling predicts patient prognosis. Clin Transl Med.

[10] Schmitt AM, Chang HY (2016). Long noncoding RNAs in cancer pathways. Cancer Cell.

[11] Iyer MK, Niknafs YS, Malik R, Singhal U, Sahu A, Hosono Y (2015). The landscape of long noncoding RNAs in the human transcriptome. Nat Genet.

[12] Derrien T, Johnson R, Bussotti G, Tanzer A, Djebali S, Tilgner H (2012). The GENCODE v7 catalog of human long noncoding RNAs: analysis of their gene structure, evolution, and expression. Genome Res.

[13] Pefanis E, Wang J, Rothschild G, Lim J, Chao J, Rabadan R (2014). Noncoding RNA transcription targets AID to divergently transcribed loci in B cells. Nature.

[14] Sun Z, Nair A, Chen X, Prodduturi N, Wang J, Kocher J-P (2017). UClncR: Ultrafast and comprehensive long non-coding RNA detection from RNA-seq. Sci Rep.

[15] Arnes L, Liu Z, Wang J, Maurer HC, Sagalovskiy I, Sanchez-Martin M (2019). Comprehensive characterisation of compartment-specific long non-coding RNAs associated with pancreatic ductal adenocarcinoma. Gut.

[16] Zerbino DR, Achuthan P, Akanni W, Amode MR, Barrell D, Bhai J (2018). Ensembl 2018. Nucleic Acids Res.

[17] O’Leary NA, Wright MW, Brister JR, Ciufo S, Haddad D, McVeigh R (2016). Reference sequence (RefSeq) database at NCBI: current status, taxonomic expansion, and functional annotation. Nucleic Acids Res.

[18] Zhou X, Yin C, Dang Y, Ye F, Zhang G (2015). Identification of the long non-coding RNA H19 in plasma as a novel biomarker for diagnosis of gastric cancer. Sci Rep.

[19] Hajjari M, Behmanesh M, Sadeghizadeh M, Zeinoddini M (2013). Up-regulation of HOTAIR long non-coding RNA in human gastric adenocarcinoma tissues. Med Oncol.

[20] Ma H-W, Xie M, Sun M, Chen T-Y, Jin R-R, Ma T-S (2017). The pseudogene derived long noncoding RNA DUXAP8 promotes gastric cancer cell proliferation and migration via epigenetically silencing PLEKHO1 expression. Oncotarget.

[21] Raveh E, Matouk IJ, Gilon M, Hochberg A (2015). The H19 Long non-coding RNA in cancer initiation, progression and metastasis—a proposed unifying theory. Mol Cancer.

[22] Zhang E, He X, Zhang C, Su J, Lu X, Si X (2018). A novel long noncoding RNA HOXC-AS3 mediates tumorigenesis of gastric cancer by binding to YBX1. Genome Biol.

[23] Hu WL, Jin L, Xu A, Wang YF, Thorne RF, Zhang XD (2018). GUARDIN is a p53-responsive long non-coding RNA that is essential for genomic stability. Nat Cell Biol.

[24] Yoon SJ, Park J, Shin Y, Choi Y, Park SW, Kang SG, et al. Deconvolution of diffuse gastric cancer and the suppression of CD34 on the BALB/c nude mice model. BMC Cancer. 2020. 10.1186/s12885-020-06814-4. 10.1186/s12885-020-06814-4 PMC7160933 32293340

[25] Ooi CH, Ivanova T, Wu J, Lee M, Tan IB, Tao J, et al. Oncogenic pathway combinations predict clinical prognosis in gastric cancer. PLoS Genet. 2009. 10.1371/journal.pgen.1000676. 10.1371/journal.pgen.1000676 PMC2748685 19798449

[26] Lei Z, Tan IB, Das K, Deng N, Zouridis H, Pattison S (2013). Identification of molecular subtypes of gastric cancer with different responses to pi3-kinase inhibitors and 5-fluorouracil. Gastroenterology.

[27] Kakiuchi M, Nishizawa T, Ueda H, Gotoh K, Tanaka A, Hayashi A (2014). Recurrent gain-of-function mutations of RHOA in diffuse-type gastric carcinoma. Nat Genet.

[28] Khan MR, Bukhari I, Khan R, Hussain HMJ, Wu M, Thorne RF, et al. TP53LNC-DB, the database of lncRNAs in the p53 signalling network. Database. 2019. 10.1093/database/bay136. 10.1093/database/bay136 PMC6323480 30624647

[29] Dugimont T, Montpellier C, Adriaenssens E, Lottin S, Dumont L, Iotsova V (1998). The H19 TATA-less promoter is efficiently repressed by wild-type tumor suppressor gene product p53. Oncogene.

[30] Hashad D, Elbanna A, Ibrahim A, Khedr G (2016). Evaluation of the role of circulating long non-coding RNA H19 as a promising novel biomarker in plasma of patients with gastric cancer. J Clin Lab Anal.

[31] Liu G, Xiang T, Wu QF, Wang WX (2016). Long noncoding RNA H19-derived miR-675 enhances proliferation and invasion via RUNX1 in gastric cancer cells. Oncol Res.

[32] Gan L, Lv L, Liao S (2019). Long non‑coding RNA H19 regulates cell growth and metastasis via the miR‑22‑3p/Snail1 axis in gastric cancer. Int J Oncol.

[33] Zhou J, Yang L, Zhong T, Mueller M, Men Y, Zhang N, et al. H19 lncRNA alters DNA methylation genome wide by regulating S-adenosylhomocysteine hydrolase. Nat Commun. 2015. 10.1038/ncomms10221. 10.1038/ncomms10221 PMC4703905 26687445

[34] Yang F, Bi J, Xue X, Zheng L, Zhi K, Hua J (2012). Up-regulated long non-coding RNA H19 contributes to proliferation of gastric cancer cells. FEBS J.

[35] Xavier-Magalhães A, Gonçalves CS, Fogli A, Lourenço T, Pojo M, Pereira B (2018). The long non-coding RNA HOTAIR is transcriptionally activated by HOXA9 and is an independent prognostic marker in patients with malignant glioma. Oncotarget.

[36] Wang KC, Yang YW, Liu B, Sanyal A, Corces-Zimmerman R, Chen Y (2011). A long noncoding RNA maintains active chromatin to coordinate homeotic gene expression. Nature.

[37] Liu A-NN, Qu H-JJ, Yu C-YY, Sun P (2018). Knockdown of LINC01614 inhibits lung adenocarcinoma cell progression by up-regulating miR-217 and down-regulating FOXP1. J Cell Mol Med.

[38] Qiu M, Xu Y, Wang J, Zhang E, Sun M, Zheng Y (2015). A novel lncRNA, LUADT1, promotes lung adenocarcinoma proliferation via the epigenetic suppression of p27. Cell Death Dis.

[39] Huang G-W, Xue Y-J, Wu Z-Y, Xu X-E, Wu J-Y, Cao H-H (2018). A three-lncRNA signature predicts overall survival and disease-free survival in patients with esophageal squamous cell carcinoma. BMC Cancer.

