# Supplementary material for: Classifying gastric cancer using FLORA reveals clinically relevant molecular subtypes and highlights LINC01614 as a biomarker for patient prognosis
Source: Oncogene. 2021 Mar 19;40(16):2898–909. doi: 10.1038/s41388-021-01743-3 (PMC8062268; doi:10.1038/s41388-021-01743-3)
Supplement: Supplementary file 1 — Supplementary Methods [file 41388_2021_1743_MOESM1_ESM.pdf]

Supplementary Materials and Methods

The FLORA toolkit

The FLORA was developed into a user-friend command line tool (http://wang-lab.ust.hk/software/Software.html), which includes several independent components:

(1) generateFilteredBams utilizes BEDTools [1] to remove reads mapped to coding regions and other custom-defined genomic locations from bam files, and reads with low mapping quality (MAPQ < 30) were removed using Samtools [2].

(2) StringTie [3] was incorporated to construct the transcriptome from the preprocessed data. Assembled transcripts in each sample were selected for merging by StringTie merge if all following criteria were satisfied: (A) longer than 200 nucleotides; (B) with expression level over 0.1 TPM and 0.1 FPKM; (C) account for over 10% of all isoforms from the same loci.

(3) filterTranscripts to identify prospective lncRNAs from the assembled transcriptome. Transcripts were selected as prospective lncRNAs by the following criteria: (A) longer than 200 nucleotides; (B) containing two or more exons; (C) coding potential score larger than 0.364 as predicted by Coding Potential Assessment Tool (CPAT) [4].

(4) AnnotateNovelLncRNA used cuffcompare [5] to compare the prospective lncRNAs with RefSeq (Release 109, GRCh38.p12), GENCODE Release 27 (GRCh38.p10) and Ensembl (GRCh38.p12) annotation, and lncRNA-expressing loci with no overlap with known genomic features were defined as novel.

(5) The functions of lncRNAs were predicted via the construction of gene co-expression network based on Spearman’s correlation coefficient. Gene Ontology (GO) enrichment analysis was performed with g-profiler [6] used the coding genes with positive and significant Spearman’s correlation coefficient (Benjamini-Hochberg adjusted *P*-value < 0.001) with the lncRNA. All the networks were visualized by Cytoscape [7].

Comparison of the fraction of reads derived from noncoding regions

To comprehensively characterize the noncoding transcriptome of GC, we reanalyzed the whole transcriptome sequencing data of 407 TCGA samples, including 375 GC and 32 tumor-adjacent samples from 380 patients (Table S1) using the FLORA pipeline. The total mapped reads in the raw bam file and bam files sequentially filtered by the generateFilteredBam to remove the protein coding genes (gene type as protein coding in GENCODE v27), other transcripts (including immunoglobin and T-cell receptor family, mitochondrial genes, miRNA, misc_RNA, pseudogenes, rRNA, ribozyme, sRNA, scRNA, scaRNA, snRNA, snoRNA and vaultRNA in GENCODE v27) and rRNAs (downloaded from RefSeq), and mapping quality (with MAPQ below 10 removed) were counted. The remaining reads are counted towards the "potential known and novel lncRNAs" that are useful in noncoding transcript assembly.

The fraction of reads derived from potential known and novel lncRNAs are compared between paired tumor and tumor-adjacent normal samples from 32 patients in TCGA using paired t-test. Among the 32 pairs, we observed that the fraction of noncoding region-derived regions was significantly higher in tumor compared to normal (P = 0.0045). Higher or similar fraction of noncoding reads was observed in tumor compared to tumor-adjacent samples, in the majority of cases. However, 5 normal samples derived from the same institute (sample ID: TCGA-HU-A4GY-11A, TCGA-HU-A4GH-11A, TCGA-HU-A4GP-11A, TCGA-HU-A4GC-11A, TCGA-HU-A4HB-11A) showed unusually higher fraction of noncoding region-derived reads (fraction ranging from 3.0% to 7.8%) than other 27 normal samples (0.9% to 2.8%), suggesting potential batch effects in these 5 samples. Thus, these 5 normal and 5 paired tumor samples are excluded from this analysis.

Gene expression calculation

The featureCount module [8] from R package 'Subread' was used to call read counts of annotated genes in GENCODE Release 27 (GRCh38.p10) and novel lncRNAs identified by FLORA, and normalized as FPKM (Fragments Per Kilobase per Million mapped fragments).

Differential expression analysis

Differential expression analysis of lncRNAs between tumor and normal samples was performed with DESeq2 [9]. Significantly upregulated or downregulated lncRNAs were selected by Benjamin-Hochberg adjusted $P < 0.01$.

Identification of LncRNA-based molecular subtype in TCGA

To identify lncRNA-based molecular subtypes, we conducted hierarchical clustering with Ward.D linkage. The distance metric was 1 – Pearson's correlation coefficient and the procedure was iterated 1,000 times with subsampling ratio of 0.8 using normalized expression of lncRNAs which were upregulated in tumor compared to normal samples. A lncRNA's expression level in tumor samples was normalized using the average expression of the lncRNA in tumor-adjacent normal samples:

$$normalized\ expression\ in\ tumor\ sample\ A = -log_{10}\left(\frac{expression\ in\ tumor\ sample\ A\ in\ FPKM}{average\ expression\ across\ normal\ samples}\right)$$

The normalized expression level was further center-normalized. The clustering of tumor samples was performed with R package 'ConsensusClusterPlus' [10]. To assess the ideal number of identified clusters, cumulative distribution functions (CDF) of consensus indexes were estimated for k (number of clusters) from 2 to 10.

Definition of lncRNA-based subtypes (L1/L2/L3) in independent cohorts

To define L1/L2/L3 subtypes in independent cohorts with only microarray data (but not RNA-seq), we developed machine learning methods to classify GC patients. As the lncRNA-based molecular subtypes (L1/L2/L3) were well defined on TCGA patients, we first performed Wilcoxon rank-sum tests to extract features (both coding and noncoding genes) that are differentially expressed among subtypes using RNA-seq data of all TCGA GC patients. The microarray platforms capture most of coding gene but only a small number of noncoding genes, limiting the capability to directly define L1/L2/L3. Therefore, for a given patient cohort characterized by the microarray platform, we will use coding feature genes to predict their noncoding-expression subtypes. In particular, three support vector machine (SVM) classifiers were built to predict whether a sample is L1, L2 or L3 respectively.

To train the L1 classifier, we respectively selected top 1,000 differentially expressed genes (DEGs) between L1 and L2, as well as top 1,000 DEGs between L1 and L3. We then conducted principal component analysis on these DEGs to reduce the feature dimension. Subsequently, Wilcoxon rank-sum tests were performed on the principal components to select the top 50 principal components enriched in the L1 subtype. Using the Z-score normalized principal components, we tuned the hyperparameters of a SVM classifier by 10-fold cross-validation in the TCGA dataset. Finally, this classifier will be applied to the independent cohort for L1 subtyping. Similarly, L2 and L3 classifiers will be trained in the same manner. To determine the final subtype of a GC case, we will run the L3 classifier first. If it was classified as L3 the procedure will stop; otherwise, the L3-negative samples will be sequentially evaluated by L2 and L1 classifiers. The predicted subtype of samples that were not identified by any of the classifiers were labeled as “Not Available” (NA).

Survival analysis

Survival analysis was performed with python package 'lifelines' [11]. The associations between the expression of lncRNAs and survival was estimated by two approaches: (1) Cox regression analysis; (2) segregating patients into high-expression and low-expression groups by different cut-offs of FPKM level and calculating the *P*-value by log-rank test, and the result with the most significant *P*-value was reported.

Copy number alterations analysis

We used a cut-off of +/− 0.3 on the segment mean (log2 transformation of the copy number) to define copy number gain/loss, where approximately 99% of all segments in normal samples were below this threshold. GISTIC 2.0 (v2.0.12) was used to identify regions of the genome that were significantly gained or deleted across a set of samples using a Q-value cut-off <0.05 [12].

Analysis of DNA methylation level of lncRNA genes

To analyze the methylation level of all annotated and novel lncRNAs, the probes on HumanMethylation450 array were assigned to a lncRNA gene if it falls within the promoter (2 kb upstream from transcriptional start site) or within the gene body of the lncRNA. For lncRNAs that contains at least one methylation probes, the methylation level of the lncRNA is represented by the probe that satisfy these criteria: (1) methylation level of the probe is negatively associated with the expression level of the lncRNA (with Spearman's correlation coefficient below 0 and *P*-value below 0.05); (2) when more than one probes are located at the promoter and gene body of a lncRNA, the probe with the strongest negative association with lncRNA expression level were selected to represent the methylation level of the lncRNA. On the contrary, if there is either no probe on the lncRNA gene or no significant negative

correlation was observed between the lncRNA expression and the methylation level of any probes on the lncRNA, the methylation level of the lncRNA gene is considered non-available.

Characterization and prioritization of oncogenic lncRNAs in GC

The 50 lncRNAs that are upregulated and associated with poor prognosis in GC are prioritized by a priority score calculated as below:

$$Score_{Priority} = Score_{Differential\ P-value} + Score_{Prognosis\ P-value} + Score_{Amplification} + Score_{GWAS} + Score_{Experimental\ Evidence}$$

where $Score_{Differential\ P-value}$ represents the normalized rank of each lncRNA in the order of descending P-value of differential expression in GC compared to normal stomach tissues; $Score_{Prognosis\ P-value}$ represents the normalized rank of each lncRNA in the order of descending P-value in associations with poor prognosis by cox regression test; $Score_{Amplification}$ represents the normalized rank of each lncRNA in the order of increasing frequency of amplification in GC; $Score_{GWAS}$ assessed if the lncRNA is in close adjacency (within 10 Mbp) with any reported SNPs that are associated with the risk of GC (from the GWAS catalog [13]); $Score_{Experimental\ Evidence}$ indicates whether the lncRNA has been experimentally validated with oncogenic functions in any cancer types.

RNA extraction, semiquantitative reverse transcription PCR, and real-time PCR analyses

Total RNA was extracted using TRIzol Reagent (Invitrogen). Complementary DNA (cDNA) was synthesized using Transcriptor Reverse Transcriptase (Roche Applied Sciences, Indianapolis, IN). Real-time PCR was performed using an SYBR Green master mixture (Roche) on LightCycler 480 Instrument. Each sample was tested in triplicate. Experiments were repeated twice. Primer sequences for *LINC01614* and *GADPH* are listed below:

Primer	Sequence
LINC01614 Forward	TCAACCAAGAGCGAAGCCAA
LINC01614 Reverse	TTGGACACAGACCCTAGCAC
GAPDH Forward	GGGAAACTGTGGCGTGAT
GAPDH Reverse	GAGTGGGTGTCGCTGTTGA

Overexpression of *LINC01614* in GC cell lines

Full-length *LINC01614* cDNA (NR_132383.1) was cloned into pEXP-RB-Mam vector (RiboBio, Guangzhou, China). The sequence of *LINC01614* cDNA insert was confirmed by sequencing. The resulting vector or control vector (empty vector) was transfected into MKN28 cells using Lipofectamine2000 Transfection Reagent (Life Technologies, Carlsbad, CA) according to the manufacturer's protocol.

LentiCRISPR for *LINC01614* knockout experiment in GC cell lines

sgRNA (GTGTAAGGTACTCAAGTGCT) targeting *LINC01614* was cloned into the lentiCRISPR vector. Following transformation, plasmids were purified and the insertion of sgRNA was confirmed by sequencing. The resulting vector or control vector (empty vector) was transfected into 293T cells with psPAX2 and pMD2.G using Lipofectamine2000 Transfection Reagent (Life Technologies, Carlsbad, CA). Lentiviral particles were produced to infect MKN28 cells. After infection with lentivirus, cells were selected with puromycin.

Colony formation assay

Cells were seeded in 48-well plates (2×10^2 cells/well) and cultured at 37°C in 5% CO_2. After 10 days, the cells were washed with PBS and stained with crystal violet. ddH_2O was used to wash the cells three times to obtain a clean background.

Cell migration assessment by wound-healing assay

Cells were cultured in 24-well plates with the density of 5×10^4 cells per well. After confluence, a wound was made across the well with a 200 μL pipette tip. The wound was photographed immediately. The cells migrated across the gap wound were observed and documented using an inverted microscope. Area of the gap was quantified using Image J. Two different areas in each well were imaged, measured and averaged in statistical analysis.

Cell proliferation assay (MTT assay)

After lentivirus infection and puromycin selection, cells were trypsinized, resuspended, seeded in a 96-well plate with a density of 2×10^3 cells/well and incubated at 37°C. At each indicated time-point, 10 μl of MTT (3-(4,5-Dimethylthiazol-2-yl)-2,5-diphenyltetrazolium bromide) was added and incubation was continued for 4 h. At the end of the incubation period, the medium was removed carefully and 50 μl of dimethylsulfoxide (DMSO) was added. The plates were agitated and the absorbance was measured at 570 nm under an absorption spectrophotometer. Two measurements were taken for each sample and the signals were averaged in statistical analysis.

Generation and analysis of RNA-seq data of GC cell lines with *LINC01614* manipulation

After knocking out *LINC01614* in human cell lines, including GES1 and MKN1, or overexpressing *LINC01614* in human cell lines, including GES1 and MGC803, total RNA was extracted with TRIzol reagent (Thermo Fisher Scientific). RNA-seq was performed by Beijing Novogene Technology on Illumina NovaSeq 6000 platform with paired-end 150 bp (PE150). 15G of raw data were generated for each sample. The raw sequencing data was mapped to human reference genome hg38 using STAR [17], and the total read pairs mapped to each gene annotated in GENCODE release 27 were obtained using featureCounts [8] in the Subread

package. The count of each gene was normalized to FPKM. The fold-change of gene expression in each cell line after *LINC01614* over-expression or CRISPR-Cas9 knock-out was calculated in contrast to the control by $fold - change = \frac{FPKM_{experiment} + c_0}{FPKM_{control} + c_0}$, where pseudo-count $c_0 = 1$ to reduce noise in low expression genes. The expression data and fold change values were provided in Table S7. The genes that are positively regulated by *LINC01614* was defined by: (1) positively correlated with *LINC01614* in TCGA GC dataset; (2) up-regulated in both GES1 and MGC803 cell lines after *LINC01614* over-expression; (3) down-regulated in both GES1 and MKN1 cell lines after *LINC01614* CRISPR-Cas9 knock-out. The genes that are negatively regulated by *LINC01614* were defined by: (1) negatively correlated with *LINC01614* in TCGA GC dataset; (2) down-regulated in both GES1 and MGC803 cell lines after *LINC01614* over-expression; (3) up-regulated in both GES1 and MKN1 cell lines after *LINC01614* CRISPR-Cas9 knock-out.

The gene list ranked by fold change was used to perform Gene Set Enrichment Analysis [18, 19] on all gene sets in the Mutation Signature Dataset v7.2 [20], and gene sets with significant associations (NOM p-val < 0.05 and FDR q-val < 0.25) in all cell lines were reported in the Table S8.

Data and code availability

The FLORA pipeline is deposited at http://wang-lab.ust.hk/software/Software.html. Expression, mutation, copy number and clinical data generated by The Cancer Genome Atlas are available at the GDC portal: https://portal.gdc.cancer.gov/. Preprocessed sequencing data are available at the Broad GDAC firehose: https://gdac.broadinstitute.org/. The targeted DNA sequencing data was downloaded from the supplementary table of the ACRG study [21], and the copy number data was downloaded from GSE62717. Correspondence and requests for materials should be addressed to J.W. (jgwang@ust.hk) and J.Y. (junyu@cuhk.edu.hk)

Supplementary materials and methods bibliography

1. Quinlan AR, Hall IM. BEDTools: a flexible suite of utilities for comparing genomic features. Bioinformatics 2010; 26(6):841–842.
2. Li H, Handsaker B, Wysoker A, Fennell T, Ruan J, Homer N et al. The Sequence Alignment/Map format and SAMtools. Bioinformatics 2009; 25(16):2078–2079.
3. Pertea M, Pertea GM, Antonescu CM, Chang T-C, Mendell JT, Salzberg SL. StringTie enables improved reconstruction of a transcriptome from RNA-seq reads. Nat. Biotechnol. 2015; 33(3):290–295.
4. Wang L, Park HJ, Dasari S, Wang S, Kocher J-P, Li W. CPAT: Coding-Potential Assessment Tool using an alignment-free logistic regression model. Nucleic Acids Res. 2013; 41(6):e74–e74.
5. Trapnell C, Williams BA, Pertea G, Mortazavi A, Kwan G, van Baren MJ et al. Transcript assembly and quantification by RNA-Seq reveals unannotated transcripts and isoform switching during cell differentiation. Nat. Biotechnol. 2010; 28(5):511–515.
6. Reimand U, Arak T, Adler P, Kolberg L, Reisberg S, Peterson H et al. g:Profiler—a web server for functional interpretation of gene lists (2016 update). Nucleic Acids Res. 2016. doi:10.1093/nar/gkw199.
7. Smoot ME, Ono K, Ruscheinski J, Wang P-L, Ideker T. Cytoscape 2.8: new features for data integration and network visualization. Bioinformatics 2011; 27(3):431–432.
8. Liao Y, Smyth GK, Shi W. featureCounts: an efficient general purpose program for assigning sequence reads to genomic features. Bioinformatics 2014; 30(7):923–930.
9. Love MI, Huber W, Anders S. Moderated estimation of fold change and dispersion for RNA-seq data with DESeq2. Genome Biol. 2014; 15(12):550.
10. Wilkerson MD, Hayes DN. ConsensusClusterPlus: a class discovery tool with confidence assessments and item tracking. Bioinformatics 2010; 26(12):1572–1573.
11. Davidson-Pilon C, Kalderstam J, Kuhn B, Fiore-Gartland A, Moneda L, Parij A et al. CamDavidsonPilon/lifelines: v0.14. 2018. doi:10.5281/ZENODO.1188309.
12. Mermel CH, Schumacher SE, Hill B, Meyerson ML, Beroukhim R, Getz G. GISTIC2.0 facilitates sensitive and confident localization of the targets of focal somatic copy-number alteration in human cancers. Genome Biol. 2011; 12(4):R41.
13. Buniello A, MacArthur JAL, Cerezo M, Harris LW, Hayhurst J, Malangone C et al. The NHGRI-EBI GWAS Catalog of published genome-wide association studies, targeted arrays and summary statistics 2019. Nucleic Acids Res. 2019; 47(D1):D1005–D1012.
14. Ernst J, Kellis M. ChromHMM: automating chromatin-state discovery and characterization. Nat. Methods 2012; 9(3):215–216.
15. Kundaje A, Meuleman W, Ernst J, Bilenky M, Yen A, Heravi-Moussavi A et al. Integrative analysis of 111 reference human epigenomes. Nature 2015; 518(7539):317–330.
16. ENCODE Project Consortium. An integrated encyclopedia of DNA elements in the human genome. Nature 2012; 489(7414):57–74.
17. Dobin A, Davis CA, Schlesinger F, Drenkow J, Zaleski C, Jha S et al. STAR: ultrafast universal RNA-seq aligner. Bioinformatics 2013; 29(1):15–21.
18. Subramanian A, Tamayo P, Mootha VK, Mukherjee S, Ebert BL, Gillette MA et al. Gene set enrichment analysis: A knowledge-based approach for interpreting genome-wide expression profiles. Proc. Natl. Acad. Sci. U. S. A. 2005; 102(43):15545–15550.
19. Mootha VK, Lindgren CM, Eriksson KF, Subramanian A, Sihag S, Lehar J et al. PGC-1α-responsive genes involved in oxidative phosphorylation are coordinately downregulated in human diabetes. Nat. Genet. 2003; 34(3):267–273.

20. Liberzon A, Birger C, Thorvaldsdóttir H, Ghandi M, Mesirov JP, Tamayo P. The Molecular Signatures Database Hallmark Gene Set Collection. Cell Syst. 2015; 1(6):417–425.
21. Cristescu R, Lee JJH, Nebozhyn M, Kim K-M, Ting JC, Wong SS et al. Molecular analysis of gastric cancer identifies subtypes associated with distinct clinical outcomes. Nat. Med. 2015; 21(5):449–456.
