# Supplementary material for: Classifying gastric cancer using FLORA reveals clinically relevant molecular subtypes and highlights LINC01614 as a biomarker for patient prognosis
Source: Oncogene. 2021 Mar 19;40(16):2898–909. doi: 10.1038/s41388-021-01743-3 (PMC8062268; doi:10.1038/s41388-021-01743-3)
Supplement: Supplementary file 2 — Supplementary Figures [file 41388_2021_1743_MOESM2_ESM.pdf]

Fig. S1

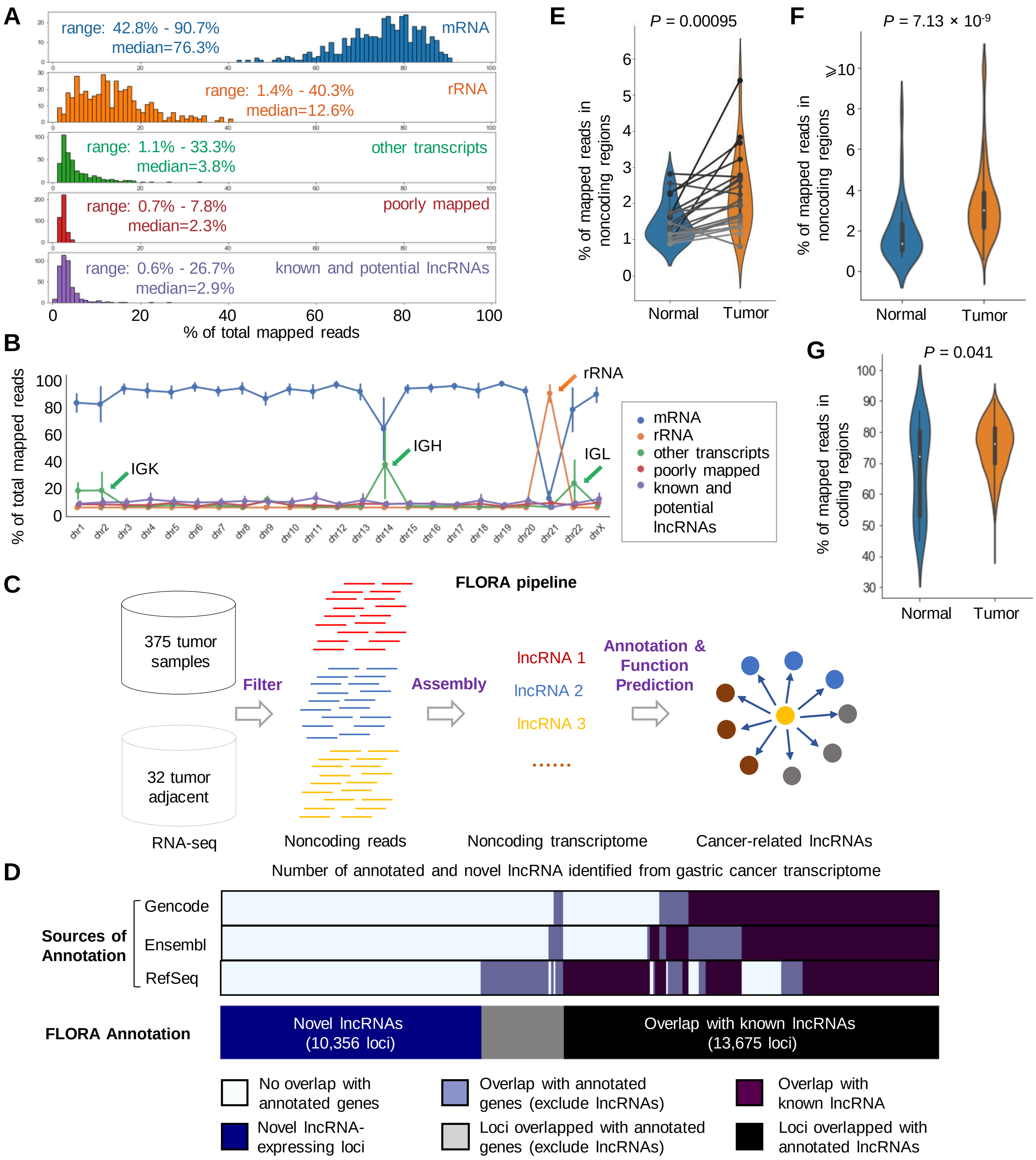

A
range: 42.8% - 90.7%
median=76.3%
mRNA
range: 1.4% - 40.3%
median=12.6%
rRNA
range: 1.1% - 33.3%
median=3.8%
other transcripts
range: 0.7% - 7.8%
median=2.3%
poorly mapped
range: 0.6% - 26.7%
median=2.9%
known and potential lncRNAs
% of total mapped reads
B
% of total mapped reads
rRNA
IGK
IGH
IGL
mRNA
rRNA
other transcripts
poorly mapped
known and potential lncRNAs
E
P = 0.00095
% of mapped reads in noncoding regions
Normal
Tumor
F
P = 7.13 × 10-9
% of mapped reads in noncoding regions
Normal
Tumor
G
P = 0.041
% of mapped reads in coding regions
Normal
Tumor
C
FLORA pipeline
375 tumor samples
32 tumor adjacent
Filter
Assembly
lncRNA 1
lncRNA 2
lncRNA 3
Annotation & Function Prediction
RNA-seq
Noncoding reads
Noncoding transcriptome
Cancer-related lncRNAs
D
Number of annotated and novel lncRNA identified from gastric cancer transcriptome
Sources of Annotation
Gencode
Ensembl
RefSeq
FLORA Annotation
Novel lncRNAs (10,356 loci)
Overlap with known lncRNAs (13,675 loci)
No overlap with annotated genes
Overlap with annotated genes (exclude lncRNAs)
Overlap with known lncRNA
Novel lncRNA-expressing loci
Loci overlapped with annotated genes (exclude lncRNAs)
Loci overlapped with annotated lncRNAs

Supplementary Figure S1. Identification of lncRNA by FLORA pipeline.

A The range and median fraction of reads from different types of transcripts in the whole transcriptome sequencing data of normal gastric samples and gastric cancer in the TCGA cohort.

B Fraction of reads mapped to different RNA species and reads with low mapping quality on each chromosome across all normal and tumor tissues. Genes that constitute large fractions of total mapped reads on several chromosomes are marked. The mean values are marked as dot, and standard derivations are marked as ticks.

C Overview of the workflow of the FLORA pipeline.

D Number of known and novel lncRNAs in TCGA GC by FLORA pipeline. The 28,507 loci encoding potential lncRNAs are represented as columns. Loci that overlap with known lncRNAs or other RNA species in Gencode, Ensembl or RefSeq data are marked by purple and light blue, respectively. 13,675 loci overlap with known lncRNAs, and 10,356 loci that do not overlap with any annotated genes are defined as novel lncRNAs.

E Fraction of reads mapped to noncoding regions of the genome in the TCGA GC patients with paired normal and tumor samples (N=27). The normal and tumor samples from the same patient are connected with lines. The p-value is calculated by two-tailed paired t-test.

F-G Fraction of reads mapped to noncoding (F) and coding regions (G) of the genome in all tumor (N=375) and normal (N=32) TCGA samples. P-values are calculated by the Wilcox's rank-sum test.

Fig. S2

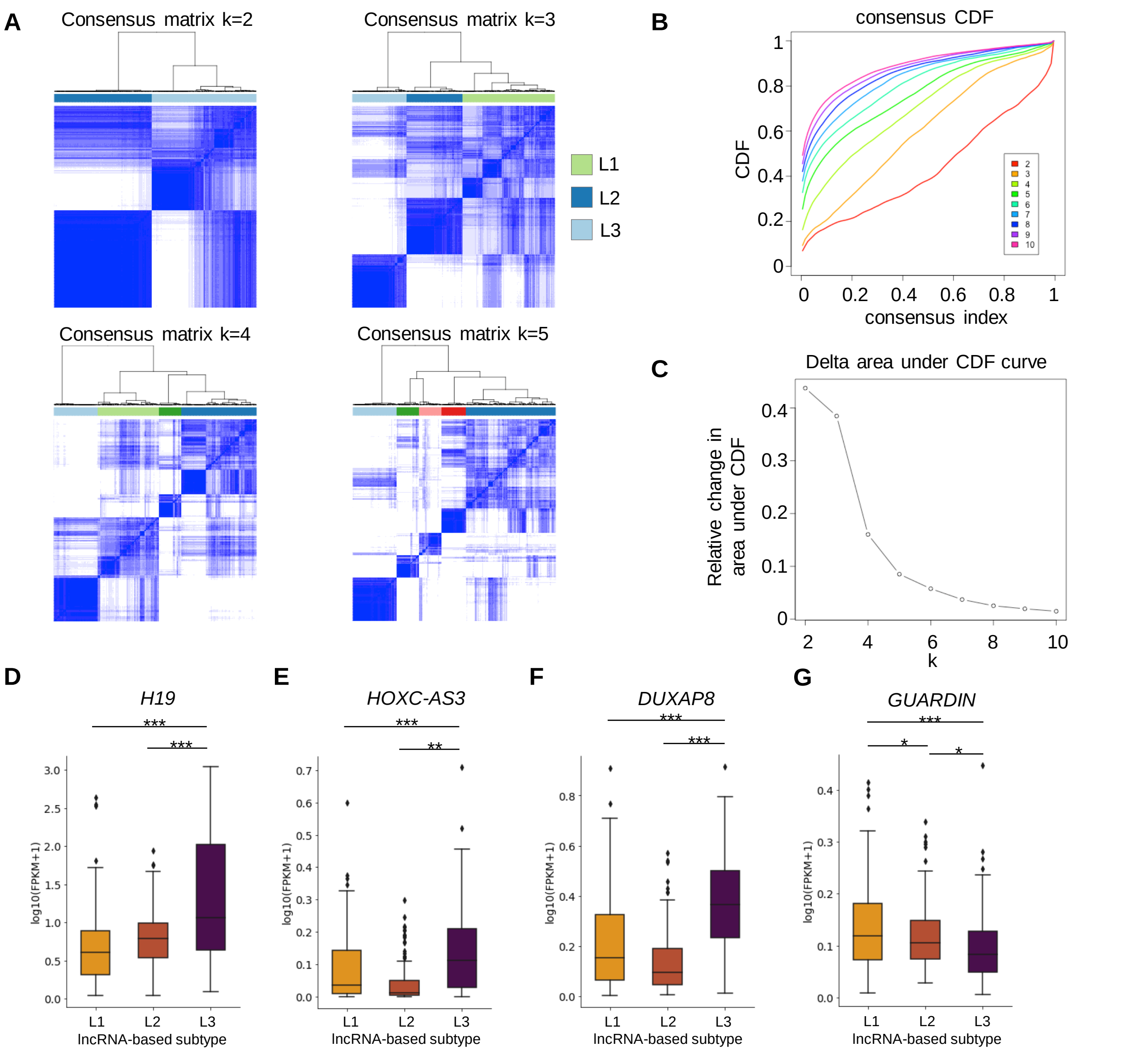

Supplementary Figure S2. Identification of lncRNA expressed-based GC subtypes.

A Consensus matrices of lncRNA expression-based clustering with the number of classes k ranging from 2 to 5.

B Cumulative distribution function (CDF) curve of consensus index for different clustering results with the number of classes k ranging from 2 to 10.

C Relative change in area under CDF curve shown in panel B.

D-G Examples of differentially expressed lncRNAs in L3 subtype compared with L1 and L2 subtypes, in GC samples from the TCGA cohort. The midline, boxes, whiskers and dots show the median, quartiles, ranges and outliers of the distribution, respectively. * $P < 0.05$, ** $P < 0.01$, *** $P < 0.001$ by unpaired two-tailed t-test.

Fig. S3

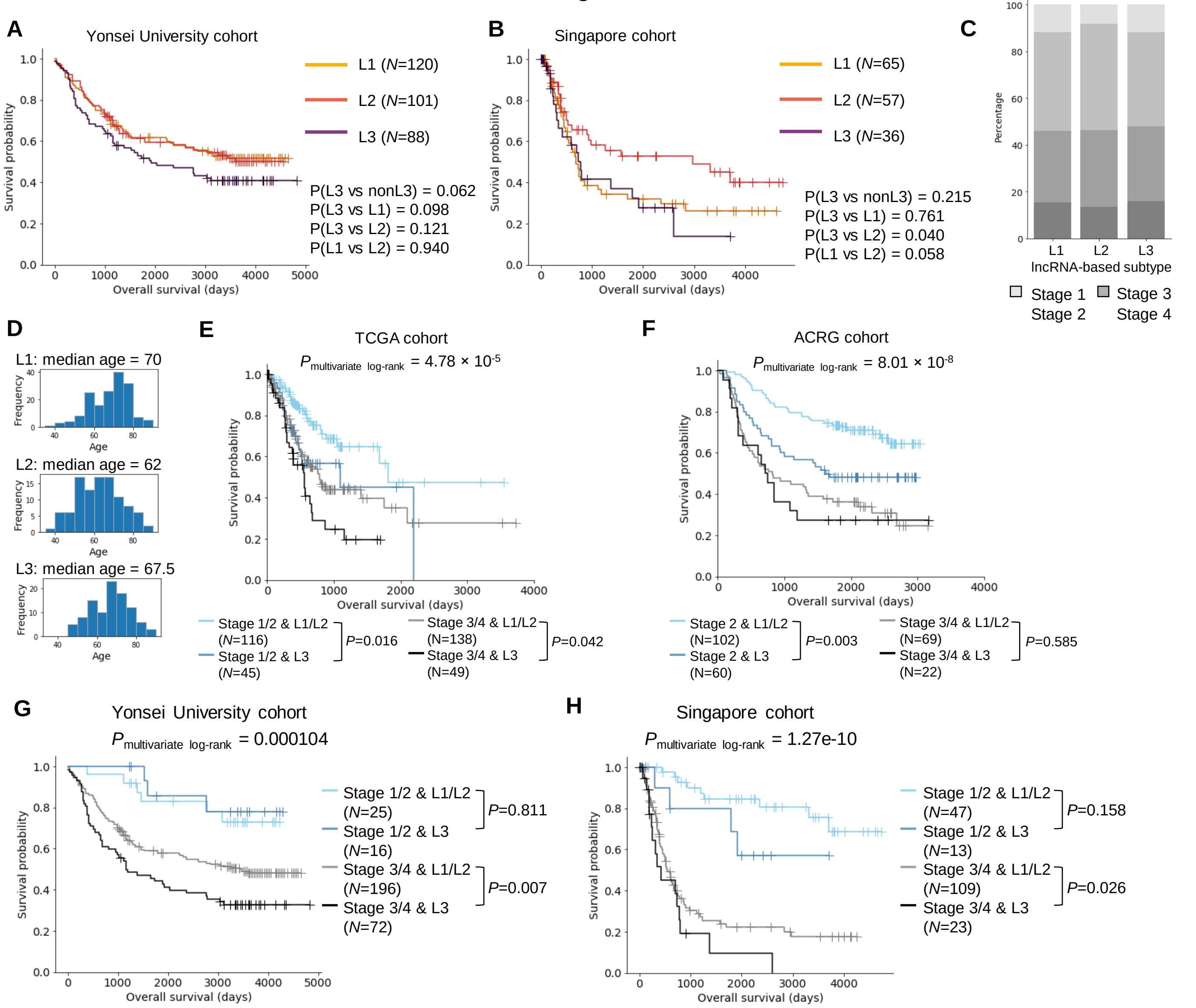

Supplementary Figure S3. Comparisons of the survival differences in different GC subtypes.

A-B Kaplan-Meier curve illustrating the overall survival for GC patients of the three subtypes from the Yonsei University (A) and Singapore (B) cohort. P-values are calculated by log-rank test.

C Distribution of tumor stages in the three lncRNA-based subtypes.

D Distribution of age at diagnosis in the three lncRNA-based subtypes.

E-H Survival probability of patients segregated by the combination of tumor staging and lncRNA-based subtypes. The *P*-value across all subgroups is calculated by multivariate log-rank test, and the *P*-value of pairwise comparison by log-rank test.

Fig. S4

A

ACRG cohort			Univariate analysis		Multivariate analysis	
Factor	Variable	Comparator	HR (95% CI)	*P*-value	HR (95% CI)	*P*-value
AJCC stage	Stage 3/4	Stage 1/2	2.37 (1.72 – 3.25)	0.0000001	2.65 (1.85 – 3.80)	0.0000001
lncRNA-based subtype	L3	L1/L2	1.44 (1.26 – 3.18)	0.043204	1.72 (1.18 – 2.51)	0.004551
Age	Age ≥ 65	Age < 65	1.94 (1.22 – 3.09)	0.007213	1.69 (1.18 – 2.42)	0.003858
Histology	Diffuse	Intestinal	1.54 (0.92 – 2.57)	0.000865	1.47 (1.14 – 1.90)	0.002800
EBV	EBV	No EBV	1.06 (0.56 – 2.01)	0.867122		

B

Yonsei University cohort			Univariate analysis		Multivariate analysis	
Factor	Variable	Comparator	HR (95% CI)	*P*-value	HR (95% CI)	*P*-value
AJCC stage	Stage 3/4	Stage 1/2	3.25 (1.65 – 6.37)	0.000615	3.41 (1.74 – 6.71)	0.000375
lncRNA-based subtype	L3	L1/L2	1.38 (0.98 – 1.93)	0.062949	1.48 (1.05 – 2.07)	0.023779
Age	Age ≥ 65	Age < 65	1.48 (1.07 – 2.04)	0.01782	1.42 (1.03 – 1.97)	0.032415

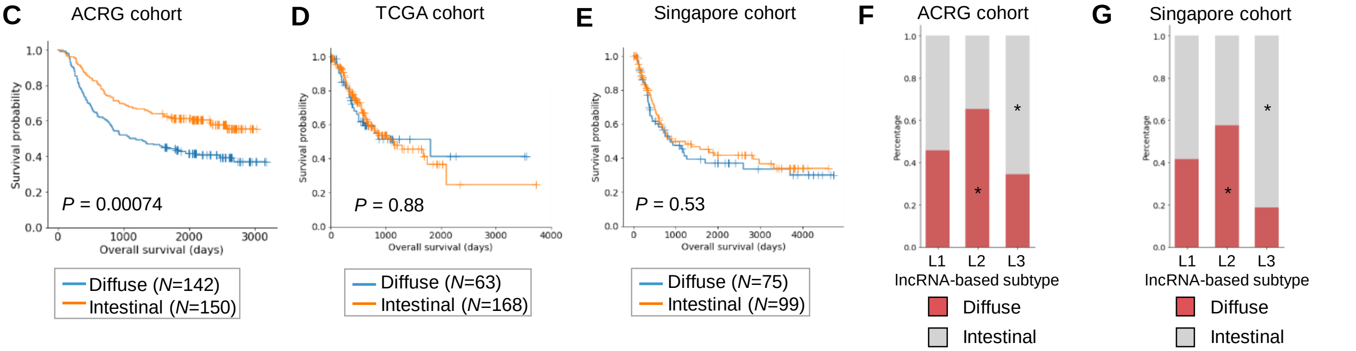

Supplementary Figure S4. The lncRNA-based subtype is an independent prognostic factor in GC.

A-B Value and 95% confidence intervals (95% CI) of the hazard ratios (HR) of different factors considered in the univariate and multivariate cox regression analysis of ACRG (A) and Yonsei University cohort (B).

C-E Survival probability of ACRG (C), TCGA (D) and Singapore cohort (E) patients with diffuse and intestinal subtype. *P*-value between subtypes are calculated by log-rank test.

F-G Distribution of diffuse and intestinal histology in the three lncRNA-based subtypes from the ACRG (F) and Singapore cohort (G). Asterisks indicate significantly enriched histological subtype in each of the three lncRNA-based subtypes by one-sided Fisher's exact test ($P < 0.05$).

Fig. S5

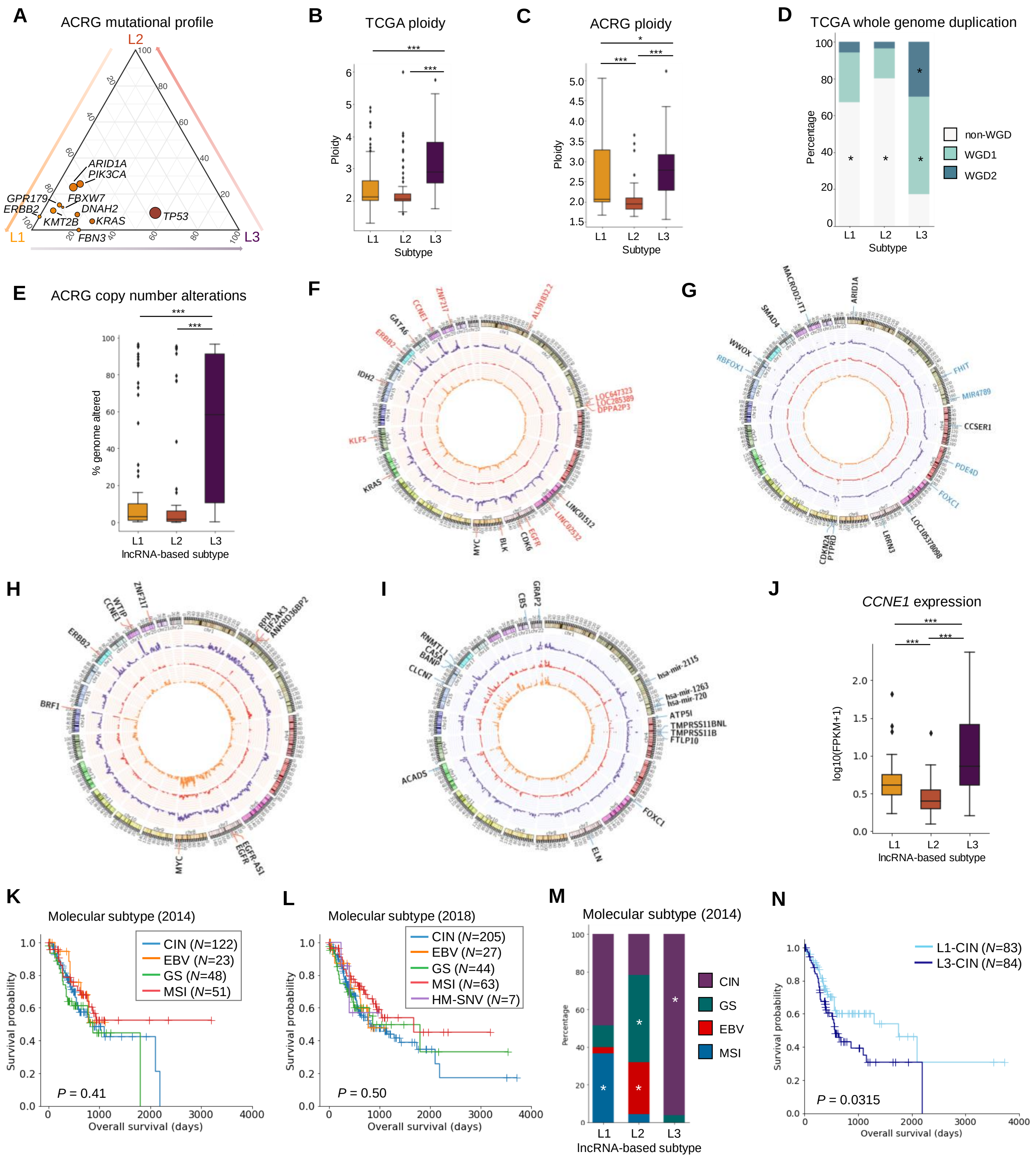
A
ACRG mutational profile
L2
L1
L3
ARID1A
PIK3CA
GPR179
FBXW7
ERBB2
DNAH2
KMT2B
KRAS
TP53
FBN3
B
TCGA ploidy
Ploidy
Subtype
C
ACRG ploidy
Ploidy
Subtype
D
TCGA whole genome duplication
Percentage
Subtype
non-WGD
WGD1
WGD2
E
ACRG copy number alterations
% genome altered
lncRNA-based subtype
F
G
H
I
J
CCNE1 expression
log10(FPKM+1)
lncRNA-based subtype
K
Molecular subtype (2014)
CIN (N=122)
EBV (N=23)
GS (N=48)
MSI (N=51)
Survival probability
Overall survival (days)
P = 0.41
L
Molecular subtype (2018)
CIN (N=205)
EBV (N=27)
GS (N=44)
MSI (N=63)
HM-SNV (N=7)
Survival probability
Overall survival (days)
P = 0.50
M
Molecular subtype (2014)
Percentage
CIN
GS
EBV
MSI
lncRNA-based subtype
N
L1-CIN (N=83)
L3-CIN (N=84)
Survival probability
Overall survival (days)
P = 0.0315

Supplementary Figure S5. Genomic alterations, copy number variations, gene expression and survival in GC patients of lncRNA-based subtypes.

A Frequency of somatic mutations which are significantly enriched in lncRNA-based subtype ($P < 0.01$ by Fisher's exact test). Each dot represents one frequently mutated gene in GC, with size proportional to its mutation frequency.

B-C Ploidy level of GC samples of L1, L2 and L3 subtypes in the TCGA (B) and ACRG cohort (C). * $P < 0.05$, *** $P < 0.001$ by Wilcox's rank-sum test.

D Percentage of L1, L2 and L3 subtype samples from TCGA with whole-genome duplications (WGD1 and WGD2) and non-WGD, defined in Liu et al. * $P < 0.05$ by Fisher's exact test.

E The fraction of genome with copy number variations in different lncRNA-based subtypes in the ACRG cohort. * $P < 0.05$, ** $P < 0.01$, *** $P < 0.001$ by Wilcox's rank-sum test.

F-G Recurrently amplified (F) and deleted (G) regions observed in lncRNA-based subtypes L1, L2 and L3 (in orange, red and purple, respectively) from the TCGA cohort. The x-axis of circles represents the chromosomal location and the y-axis represents the G-score of each location (ranging from 0-1.5 for amplifications and 0-1 for deletions). Genes located at significantly amplified/deleted peaks were marked, with genes that are more frequently amplified or deleted in L3 highlighted in red and blue, respectively.

H-I Recurrently amplified (H) and deleted (I) regions observed in lncRNA-based subtypes L1, L2 and L3 (in orange, red and purple, respectively) from the ACRG cohort. The x-axis of circles represents the chromosomal location and the y-axis represents the G-score of each location (ranging from 0-2.0 for amplifications and 0-2.0 for deletions).

J Over-expression of *CCNE1* was observed in L3 subtype compared with L1 and L2. *** $P < 0.001$ by Wilcox's rank-sum test.

K-L Survival probability of TCGA GC patients in different molecular subtypes defined by Bass et al., (K) and Liu et al., (L). *P*-value is calculated by multivariant log-rank test.

M Distribution of TCGA molecular subtypes (defined by Bass et al., 2014) in the three lncRNA-based subtypes. * $P < 0.05$ by Fisher's exact test.

N Survival probability of L1-CIN and L3-CIN subgroups. *P*-value is calculated by log-rank test.

Fig. S6

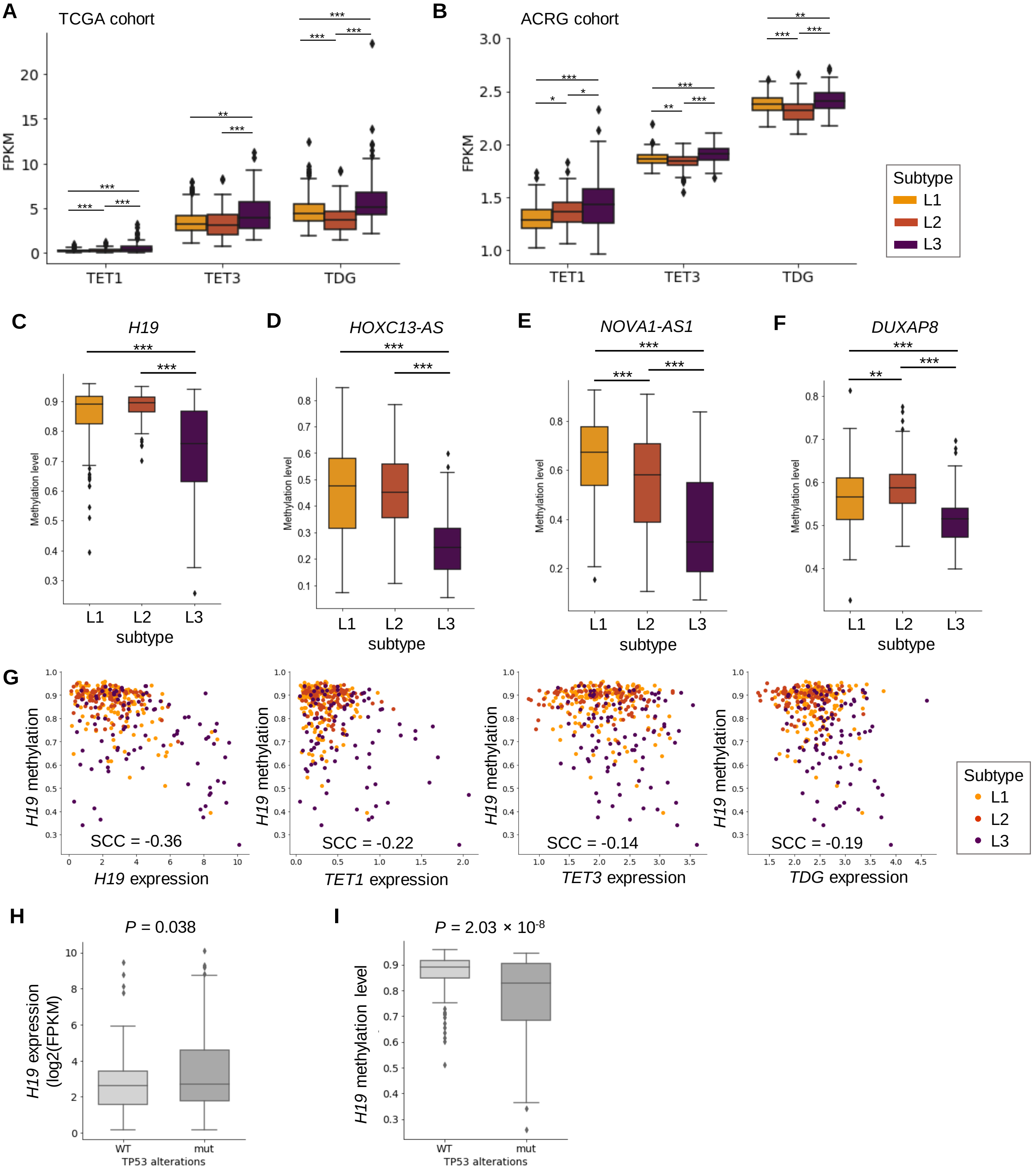
A
TCGA cohort
FPKM
TET1
TET3
TDG
B
ACRG cohort
Subtype
L1
L2
L3
C
H19
D
HOXC13-AS
E
NOVA1-AS1
F
DUXAP8
Methylation level
subtype
G
H19 methylation
SCC = -0.36
SCC = -0.22
SCC = -0.14
SCC = -0.19
H19 expression
TET1 expression
TET3 expression
TDG expression
H
P = 0.038
H19 expression (log2(FPKM))
WT
mut
TP53 alterations
I
P = 2.03 × 10-8
H19 methylation level

Supplementary Figure S6. DNA hypomethylation and *TP53* mutations in L3 subtype are associated lncRNA overexpression.

A-B Expression of *TET1*, *TET3* and *TDG* in lncRNA-based GC subtypes in the TCGA (A) and ACRG (B) cohorts.

C-F Hypomethylation of lncRNA species that are over-expressed in L3. The midline, boxes, whiskers and dots show the median, quartiles, ranges and outliers of the distribution, respectively.

G *H19* methylation level is anti-correlated with the expression level of *H19*, *TET1*, *TET3* and *TDG*. Probe cg11716026 was selected to represent *H19* gene methylation level. Each dot represents one GC sample in the TCGA dataset. GC samples of lncRNA expression-based subtype L1, L2 and L3 are shown in yellow, red and purple, respectively.

H-I Level of *H19* expression (H) and methylation (I) in TCGA GC samples with no *TP53* alterations and with *TP53* mutations and/or deletions. The midline, boxes, whiskers and dots show the median, quartiles, ranges and outliers of the distribution, respectively. * $P < 0.05$, ** $P < 0.01$, *** $P < 0.001$ by two-tailed unpaired t-test.

Fig. S7

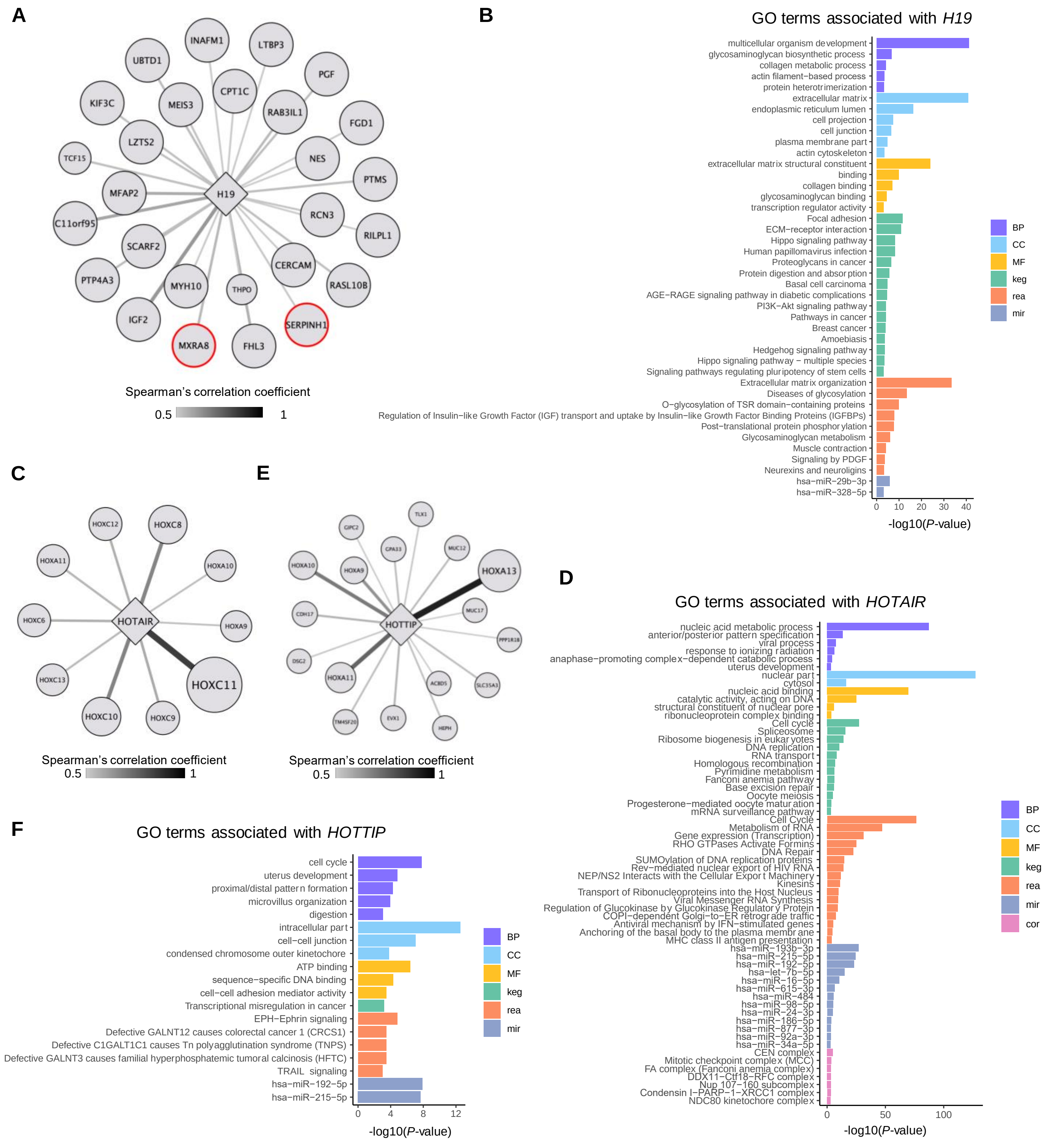

A
INAFM1
LTBP3
UBTD1
PGF
CPT1C
KIF3C
MEIS3
RAB3IL1
FGD1
LZTS2
NES
TCF15
PTMS
MFAP2
H19
RCN3
C11orf95
RILPL1
SCARF2
CERCAM
PTP4A3
MYH10
THPO
RASL10B
IGF2
SERPINH1
MXRA8
FHL3
Spearman's correlation coefficient
0.5
1
B
GO terms associated with H19
multicellular organism development
glycosaminoglycan biosynthetic process
collagen metabolic process
actin filament−based process
protein heterotrimerization
extracellular matrix
endoplasmic reticulum lumen
cell projection
cell junction
plasma membrane part
actin cytoskeleton
extracellular matrix structural constituent
binding
collagen binding
glycosaminoglycan binding
transcription regulator activity
Focal adhesion
ECM−receptor interaction
Hippo signaling pathway
Human papillomavirus infection
Proteoglycans in cancer
Protein digestion and absorption
Basal cell carcinoma
AGE−RAGE signaling pathway in diabetic complications
PI3K−Akt signaling pathway
Pathways in cancer
Breast cancer
Amoebiasis
Hedgehog signaling pathway
Hippo signaling pathway − multiple species
Signaling pathways regulating pluripotency of stem cells
Extracellular matrix organization
Diseases of glycosylation
O−glycosylation of TSR domain−containing proteins
Regulation of Insulin−like Growth Factor (IGF) transport and uptake by Insulin−like Growth Factor Binding Proteins (IGFBPs)
Post−translational protein phosphorylation
Glycosaminoglycan metabolism
Muscle contraction
Signaling by PDGF
Neurexins and neuroligins
hsa−miR−29b−3p
hsa−miR−328−5p
0
10
20
30
40
-log10(P-value)
BP
CC
MF
keg
rea
mir
C
HOXC12
HOXC8
HOXA11
HOXA10
HOXC6
HOTAIR
HOXA9
HOXC13
HOXC11
HOXC10
HOXC9
Spearman's correlation coefficient
0.5
1
E
TLX1
GPC2
GPA33
MUC12
HOXA10
HOXA9
HOXA13
MUC17
CDH17
HOTTIP
DSG2
HOXA11
TM4SF20
EVX1
HEPH
Spearman's correlation coefficient
0.5
1
D
GO terms associated with HOTAIR
nucleic acid metabolic process
anterior/posterior pattern specification
viral process
response to ionizing radiation
anaphase−promoting complex−dependent catabolic process
uterus development
nuclear part
cytosol
nucleic acid binding
catalytic activity, acting on DNA
structural constituent of nuclear pore
ribonucleoprotein complex binding
Cell cycle
Spliceosome
Ribosome biogenesis in eukaryotes
DNA replication
RNA transport
Homologous recombination
Pyrimidine metabolism
Fanconi anemia pathway
Base excision repair
Oocyte meiosis
Progesterone−mediated oocyte maturation
mRNA surveillance pathway
Cell Cycle
Metabolism of RNA
Gene expression (Transcription)
RHO GTPases Activate Formins
DNA Repair
SUMOylation of DNA replication proteins
Rev−mediated nuclear export of HIV RNA
NEP/NS2 Interacts with the Cellular Export Machinery
Kinesins
Transport of Ribonucleoproteins into the Host Nucleus
Viral Messenger RNA Synthesis
Regulation of Glucokinase by Glucokinase Regulatory Protein
COPI−dependent Golgi−to−ER retrograde traffic
Antiviral mechanism by IFN−stimulated genes
Anchoring of the basal body to the plasma membrane
MHC class II antigen presentation
hsa−miR−193b−3p
hsa−miR−215−5p
hsa−miR−192−5p
hsa−let−7b−5p
hsa−miR−16−5p
hsa−miR−615−3p
hsa−miR−484
hsa−miR−98−5p
hsa−miR−24−3p
hsa−miR−186−5p
hsa−miR−877−3p
hsa−miR−92a−3p
hsa−miR−34a−5p
CEN complex
Mitotic checkpoint complex (MCC)
FA complex (Fanconi anemia complex)
DDX11−Ctf18−RFC complex
Nup 107−160 subcomplex
Condensin I−PARP−1−XRCC1 complex
NDC80 kinetochore complex
0
50
100
-log10(P-value)
BP
CC
MF
keg
rea
mir
cor
F
GO terms associated with HOTTIP
cell cycle
uterus development
proximal/distal pattern formation
microvillus organization
digestion
intracellular part
cell−cell junction
condensed chromosome outer kinetochore
ATP binding
sequence−specific DNA binding
cell−cell adhesion mediator activity
Transcriptional misregulation in cancer
EPH−Ephrin signaling
Defective GALNT12 causes colorectal cancer 1 (CRCS1)
Defective C1GALT1C1 causes Tn polyagglutination syndrome (TNPS)
Defective GALNT3 causes familial hyperphosphatemic tumoral calcinosis (HFTC)
TRAIL signaling
hsa−miR−192−5p
hsa−miR−215−5p
0
4
8
12
-log10(P-value)
BP
CC
MF
keg
rea
mir

Supplementary Figure S7. Functional prediction and experimental validation of oncogenic lncRNAs.
A,C,E Co-expression network of *H19* (A), *HOTAIR* (C) and *HOTTIP* (E) in GC. The genes with Spearman's correlation coefficient above 0.5 are showed in the network. Node size is proportional to –log10(*P*-value) of Spearman's correlation coefficient. The edge color represents the Spearman's correlation coefficient between the expression of lncRNA and its co-expressed genes.
B,D,F GO terms associated with *H19* (B), *HOTAIR* (D) and *HOTTIP* (F). The color represents three sub-ontologies of GO terms (BP: biological process; CC: cellular component; MF: molecular function; keg: KEGG pathways; rea: reactome; mir: miRNA targets).

Fig. S8

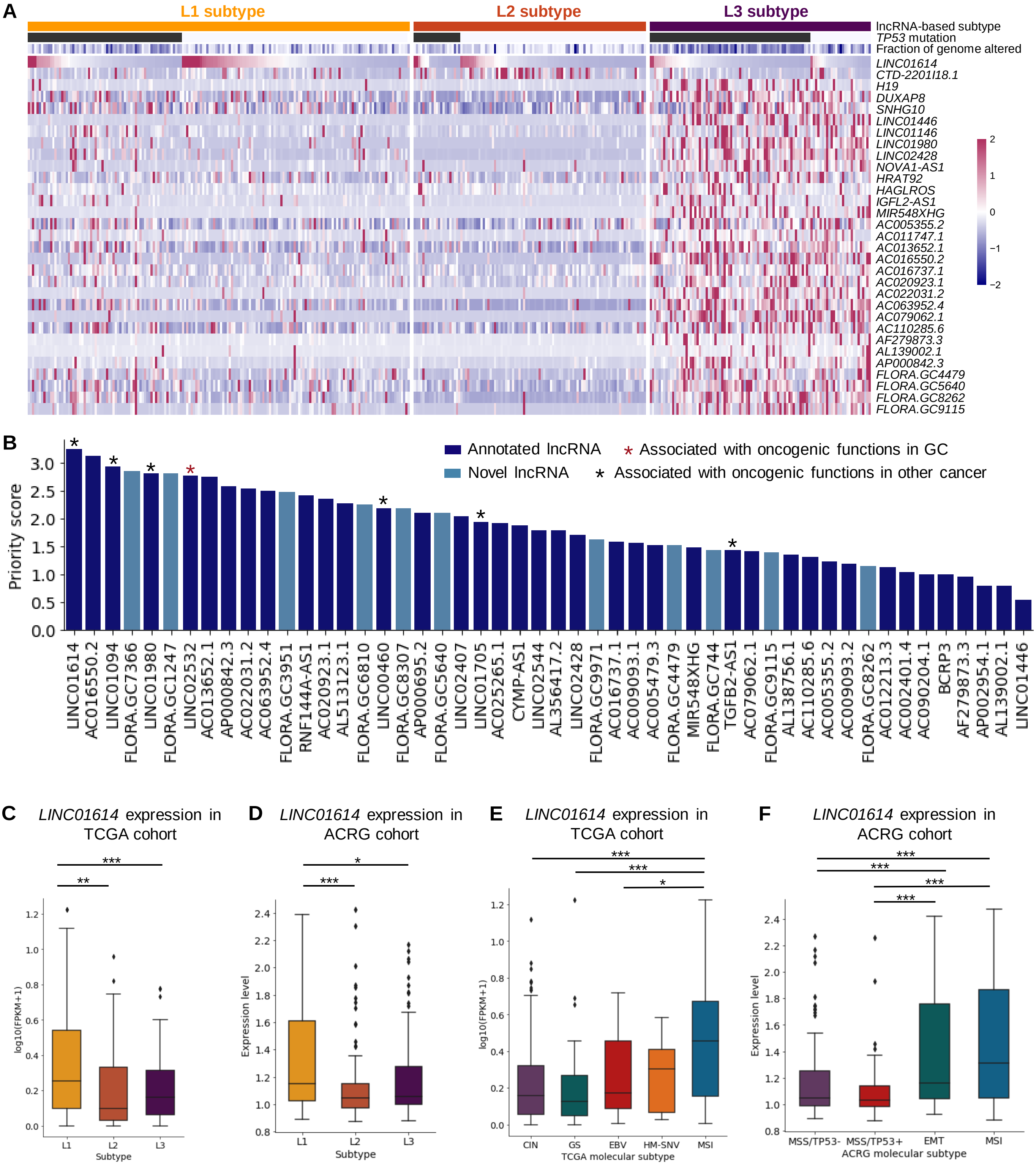

A
L1 subtype
L2 subtype
L3 subtype
lncRNA-based subtype
TP53 mutation
Fraction of genome altered
LINC01614
CTD-2201I18.1
H19
DUXAP8
SNHG10
LINC01446
LINC01146
LINC01980
LINC02428
NOVA1-AS1
HRAT92
HAGLROS
IGFL2-AS1
MIR548XHG
AC005355.2
AC011747.1
AC013652.1
AC016550.2
AC016737.1
AC020923.1
AC022031.2
AC063952.4
AC079062.1
AC110285.6
AF279873.3
AL139002.1
AP000842.3
FLORA.GC4479
FLORA.GC5640
FLORA.GC8262
FLORA.GC9115
2
1
0
−1
−2
B
Priority score
Annotated lncRNA
Novel lncRNA
Associated with oncogenic functions in GC
Associated with oncogenic functions in other cancer
LINC01614
AC016550.2
LINC01094
FLORA.GC7366
LINC01980
FLORA.GC1247
LINC02532
AC013652.1
AP000842.3
AC022031.2
AC063952.4
FLORA.GC3951
RNF144A-AS1
AC020923.1
AL513123.1
FLORA.GC6810
LINC00460
FLORA.GC8307
AP000695.2
FLORA.GC5640
LINC02407
LINC01705
AC025265.1
CYMP-AS1
LINC02544
AL356417.2
LINC02428
FLORA.GC9971
AC016737.1
AC009093.1
AC005479.3
FLORA.GC4479
MIR548XHG
FLORA.GC744
TGFB2-AS1
AC079062.1
FLORA.GC9115
AL138756.1
AC110285.6
AC005355.2
AC009093.2
FLORA.GC8262
AC012213.3
AC002401.4
AC090204.1
BCRP3
AF279873.3
AP002954.1
AL139002.1
LINC01446
C
LINC01614 expression in TCGA cohort
log10(FPKM+1)
Subtype
L1
L2
L3
D
LINC01614 expression in ACRG cohort
Expression level
Subtype
L1
L2
L3
E
LINC01614 expression in TCGA cohort
log10(FPKM+1)
TCGA molecular subtype
CIN
GS
EBV
HM-SNV
MSI
F
LINC01614 expression in ACRG cohort
Expression level
ACRG molecular subtype
MSS/TP53-
MSS/TP53+
EMT
MSI

Supplementary Figure S8. Expression of *LINC01614* in different subtypes of GC.

A Heatmap showing the molecular characteristics of the lncRNA expression-based subtypes, including prevalence of *TP53* mutations, fraction of genome with copy number alterations and expression of several subtype-specific lncRNAs.

B Prioritization of 50 GC-specific survival-related lncRNAs in GC. The annotated lncRNAs and novel lncRNAs are colored navy and blue, respectively. Asterisks indicate lncRNAs with experimental evidence of oncogenic functions.

C-D Expression of *LINC0161*4 in lncRNA expression-based subtypes in the TCGA (B) and ACRG cohort (C).

E-F Expression of *LINC01614* in molecular subtypes defined by the TCGA study (E) and by the ACRG cohort (F). * $P < 0.05$, ** $P < 0.01$, *** $P < 0.001$ by two-tailed unpaired t-test.

Fig. S9

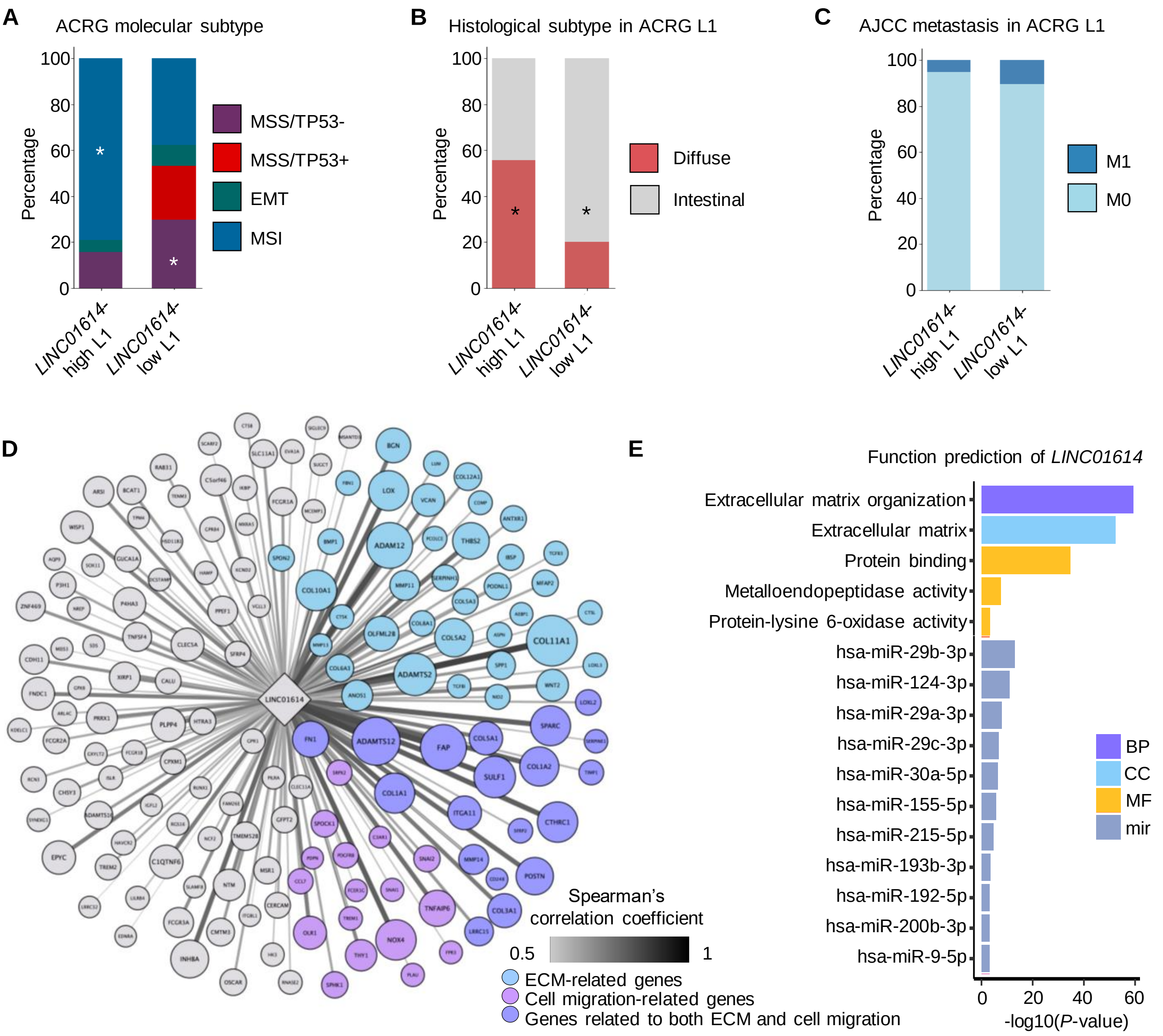

Supplementary Figure S9. Characteristic of *LINC01614*-high L1 tumor and functional prediction of *LINC01614* based on co-expression analysis in TCGA dataset.

A-C Distribution of ACRG *LINC01614*-high L1 and *LINC01614*-low L1 subgroups in molecular subtypes defined by the ACRG cohort (A), histological subtypes (B) and AJCC metastasis status (C). * $P < 0.05$ by Fisher's exact test.

D Co-expression network of *LINC01614*. Genes with Spearman's correlation coefficient > 0.5 were selected for the network visualization. Node size represents mutual information between the expression of *LINC01614* and the gene in the circle, while edge color represents the spearman correlation coefficient. Genes related to extracellular matrix (ECM), cell migration or both are colored blue, purple and orchid, respectively. **E** GO terms enriched in the co-expression network of *LINC01614* (BP: biological process; CC: cellular component; MF: molecular function; mir: miRNA targets)..

Fig. S10

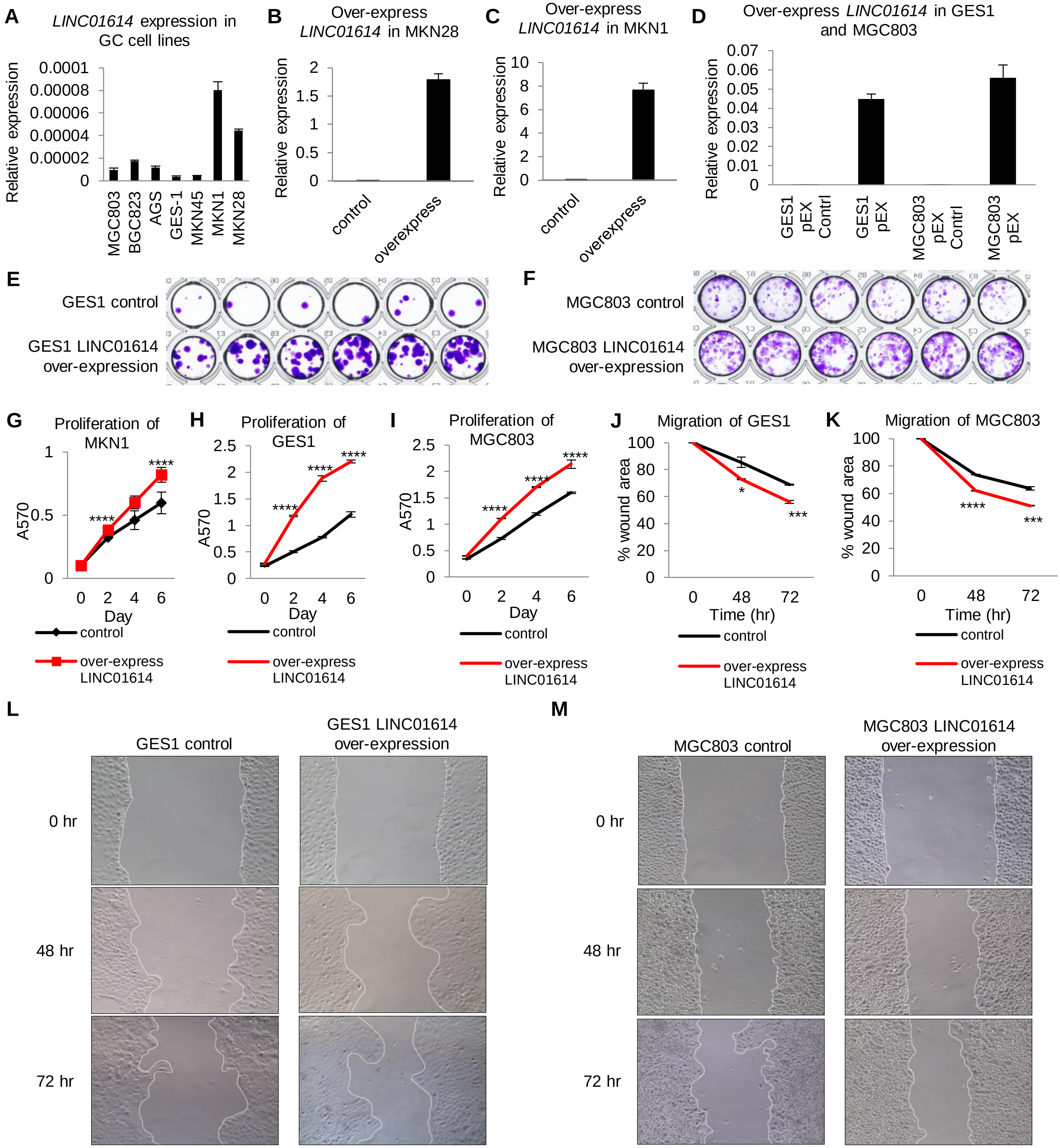
A
LINC01614 expression in GC cell lines
Relative expression
0.0001
0.00008
0.00006
0.00004
0.00002
0
MGC803
BGC823
AGS
GES-1
MKN45
MKN1
MKN28
B
Over-express LINC01614 in MKN28
Relative expression
control
overexpress
C
Over-express LINC01614 in MKN1
Relative expression
control
overexpress
D
Over-express LINC01614 in GES1 and MGC803
Relative expression
GES1 pEX Contrl
GES1 pEX
MGC803 pEX Contrl
MGC803 pEX
E
GES1 control
GES1 LINC01614 over-expression
F
MGC803 control
MGC803 LINC01614 over-expression
G
Proliferation of MKN1
A570
Day
control
over-express LINC01614
H
Proliferation of GES1
A570
Day
control
over-express LINC01614
I
Proliferation of MGC803
A570
Day
control
over-express LINC01614
J
Migration of GES1
% wound area
Time (hr)
control
over-express LINC01614
K
Migration of MGC803
% wound area
Time (hr)
control
over-express LINC01614
L
GES1 control
GES1 LINC01614 over-expression
0 hr
48 hr
72 hr
M
MGC803 control
MGC803 LINC01614 over-expression
0 hr
48 hr
72 hr

Supplementary Figure S10. Over-expression of *LINC01614* in GC cell lines promotes proliferation and migration.
A Validation of *LINC01614* expression in normal (GES1) and gastric cancer cell lines by semi-quantitative PCR.
B-D Validation of *LIN01614* over-expression in MKN28 (B), MKN1 (C), GES1 and MGC803 (D) cell lines by qPCR.
E-F Colony formation in GES1 (E) and MGC803 (F) cell lines with and without LINC01614 over-expression.
G-I Proliferation rate under *LIN01614* over-expression (red) and control (black) in MKN1 (G), GES1 (H) and MGC803 (I) cell lines.
J-K Wound healing rate under *LIN01614* over-expression (red) and control (black) in GES1 (J) and MGC803 (K) cell lines.
L-M Representative images of wound healing assay in GES1 (L) and MGC803 (M) cell lines with *LINC01614* over-expression and control.
In all experiments, three biological replicates were performed for each group. Data are represented as mean ± SD. * $P < 0.05$; ** $P < 0.01$; *** $P < 0.001$; **** $P < 0.0001$ by two-tailed unpaired t-test.

Fig. S11

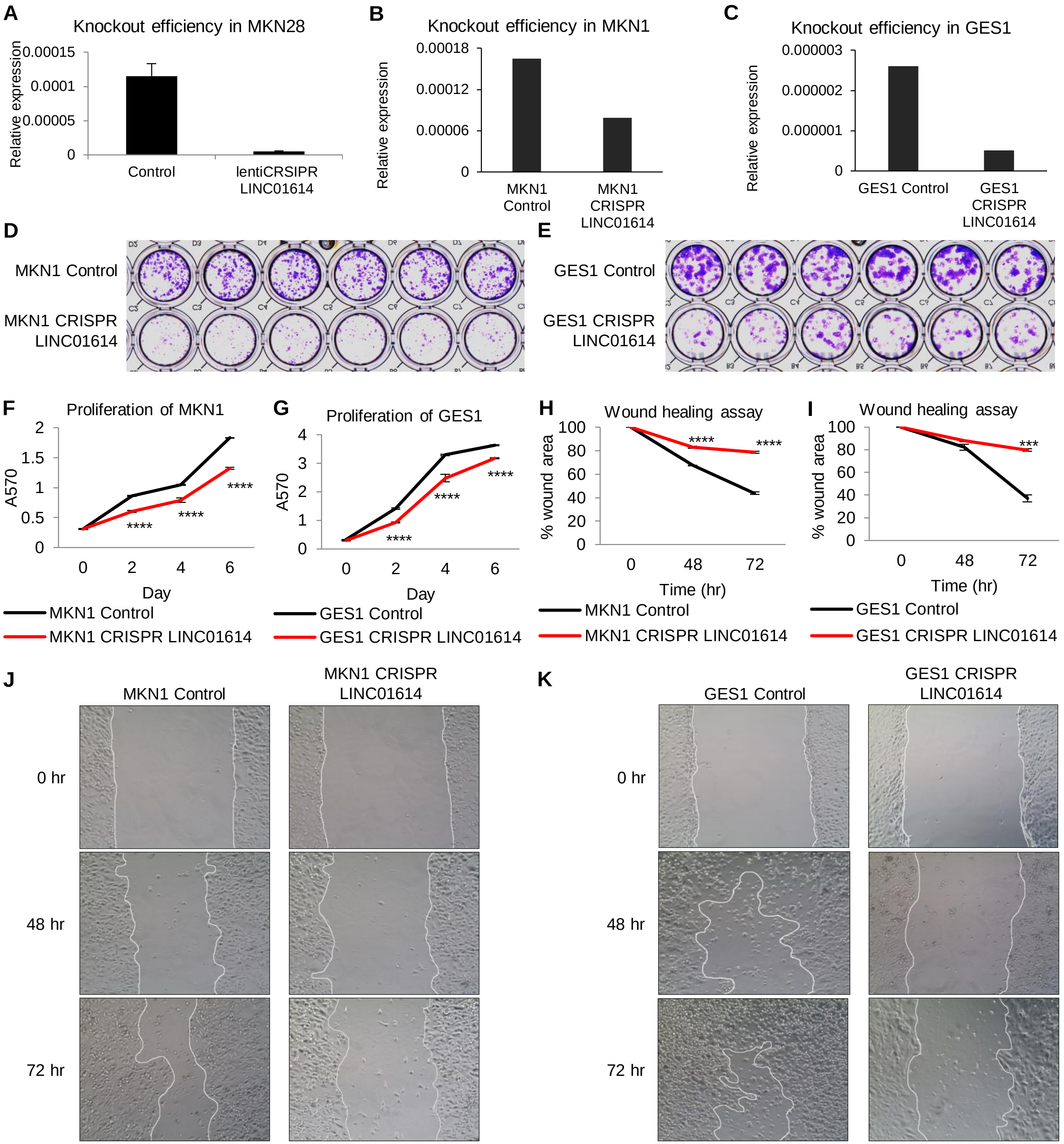
A
Knockout efficiency in MKN28
Relative expression
0.00015
0.0001
0.00005
0
Control
lentiCRSIPR LINC01614
B
Knockout efficiency in MKN1
Relative expression
0.00018
0.00012
0.00006
0
MKN1 Control
MKN1 CRISPR LINC01614
C
Knockout efficiency in GES1
Relative expression
0.000003
0.000002
0.000001
0
GES1 Control
GES1 CRISPR LINC01614
D
MKN1 Control
MKN1 CRISPR LINC01614
E
GES1 Control
GES1 CRISPR LINC01614
F
Proliferation of MKN1
A570
Day
****
MKN1 Control
MKN1 CRISPR LINC01614
G
Proliferation of GES1
A570
Day
****
GES1 Control
GES1 CRISPR LINC01614
H
Wound healing assay
% wound area
Time (hr)
****
MKN1 Control
MKN1 CRISPR LINC01614
I
Wound healing assay
% wound area
Time (hr)
***
GES1 Control
GES1 CRISPR LINC01614
J
MKN1 Control
MKN1 CRISPR LINC01614
0 hr
48 hr
72 hr
K
GES1 Control
GES1 CRISPR LINC01614
0 hr
48 hr
72 hr

Supplementary Figure S11. Experimental validation of *LINC01614* functions in driving GC cell proliferation and migration.
A-C Validation of effective CRISPR-Cas9 knockout of *LIN01614* and reduced expression in MKN28 (A), MKN1 (B) and GES1 (C) cell lines by qPCR.
D-E Colony formation in MKN1 (E) and GES1 (F) cell lines with and without CRISPR-Cas9 knockout of *LIN01614*.
F-G Proliferation rate under CRISPR-Cas9 knockout of *LIN01614* (red) and control (black) in MKN1 (F) and GES1 (G) cell lines.
H-I Wound healing rate under CRISPR-Cas9 knockout of *LIN01614* (red) and control (black) in MKN1 (H) and GES1 (I) cell lines.
J-K Representative images of wound healing assay in MKN1 (L) and GES1 (M) cell lines with CRISPR-Cas9 knockout of *LIN01614* and control.
In all experiments, three biological replicates were performed for each group. Data are represented as mean ± SD. * $P < 0.05$; ** $P < 0.01$; *** $P < 0.001$; **** $P < 0.0001$ by two-tailed unpaired t-test.

Fig. S12

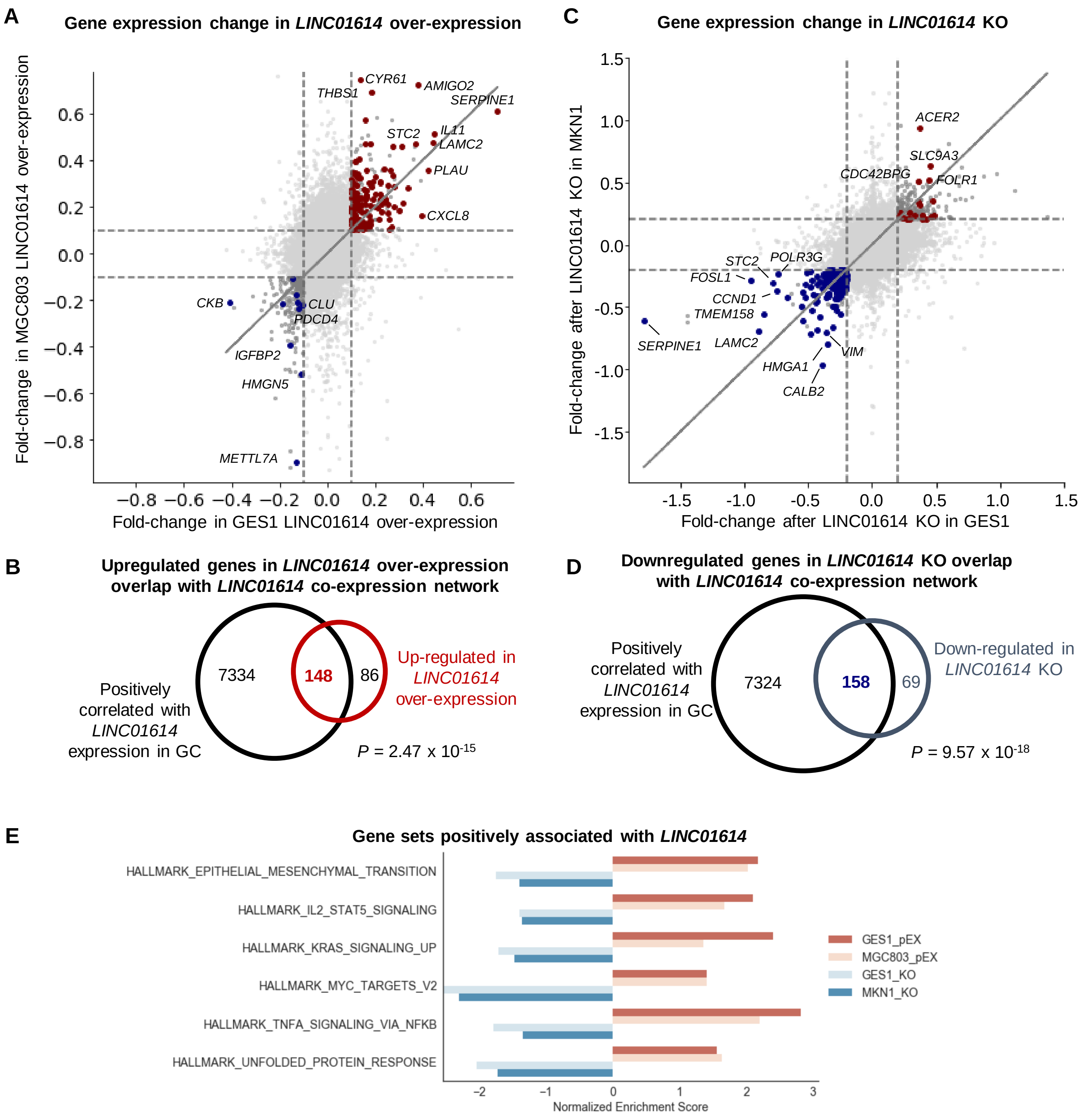
A
Gene expression change in LINC01614 over-expression
Fold-change in MGC803 LINC01614 over-expression
Fold-change in GES1 LINC01614 over-expression
0.6
0.4
0.2
0.0
−0.2
−0.4
−0.6
−0.8
0.0
0.2
0.4
0.6
CYR61
AMIGO2
THBS1
SERPINE1
STC2
IL11
LAMC2
PLAU
CXCL8
CKB
CLU
PDCD4
IGFBP2
HMGN5
METTL7A
C
Gene expression change in LINC01614 KO
Fold-change after LINC01614 KO in MKN1
Fold-change after LINC01614 KO in GES1
1.5
1.0
0.5
0.0
-0.5
-1.0
-1.5
ACER2
SLC9A3
CDC42BPG
FOLR1
STC2
POLR3G
FOSL1
CCND1
TMEM158
SERPINE1
LAMC2
VIM
HMGA1
CALB2
B
Upregulated genes in LINC01614 over-expression overlap with LINC01614 co-expression network
7334
148
86
Up-regulated in LINC01614 over-expression
Positively correlated with LINC01614 expression in GC
P = 2.47 x 10-15
D
Downregulated genes in LINC01614 KO overlap with LINC01614 co-expression network
Positively correlated with LINC01614 expression in GC
7324
158
69
Down-regulated in LINC01614 KO
P = 9.57 x 10-18
E
Gene sets positively associated with LINC01614
HALLMARK_EPITHELIAL_MESENCHYMAL_TRANSITION
HALLMARK_IL2_STAT5_SIGNALING
HALLMARK_KRAS_SIGNALING_UP
HALLMARK_MYC_TARGETS_V2
HALLMARK_TNFA_SIGNALING_VIA_NFKB
HALLMARK_UNFOLDED_PROTEIN_RESPONSE
−2
−1
0
1
2
3
Normalized Enrichment Score
GES1_pEX
MGC803_pEX
GES1_KO
MKN1_KO

Supplementary Figure S12. Analysis of transcriptome-wide changes in GC cell lines after *LINC01614* over-expression or CRISPR-Cas9 knockout.

A-B Fold change of gene expression in GC cell lines after *LINC01614* over-expression (A) or CRISPR-Cas9 knockout (KO) (B) compared to the controls.

C-D Overlap between the number of genes that are positively associated with *LINC01614* by coexpression analysis and the genes that are strongly affected by *LINC01614* over-expression (C) or *LINC01614* KO (D).

E Cancer hallmark gene sets that are significantly altered in all *LINC01614* over-expression and KO experiments (NOM p-value < 0.05 and FDR q-value < 0.25). The x-axis shows the normalized enrichment score by Gene Set Enrichment Analysis of each experiment.
